# Configurationally Stepping Confinement Achieved Tunable Chiral Near‐Infrared Luminescence Supramolecular Phenothiazine Organic Framework

**DOI:** 10.1002/advs.202408107

**Published:** 2024-09-20

**Authors:** Jie Yu, Jie Niu, Xiufang Xu, Yu Liu

**Affiliations:** ^1^ College of Chemistry State Key Laboratory of Elemento‐Organic Chemistry Nankai University Tianjin 300071 P. R. China

**Keywords:** chiral supramolecular organic framework, cucurbituril, Near‐Infrared luminescence, phenothiazine, thermally responsive

## Abstract

Herein, thermally responsive reversible chiral supramolecules are reported, constructed by the chirality transfer from tripeptides to achiral network supramolecular organic frameworks (SOF) based on configurationally stepping confinement, which displayed not only highly selective reversible chirality transfer but also efficient enhanced near‐infrared (NIR) luminescence. Taking advantage of macrocyclic confinement, CB[8] separately encapsulated two kinds of tetracationic bis(phenothiazines) derivatives (G1, G2) at 2:1 stoichiometric to form organic 2D SOFs, efficiently enhancing 12.6 fold NIR luminescence and blueshifted from 705 to 680 nm for G1, and redshifted from 695 to 710 nm for G2, respectively. Uncommonly, the tripeptide coassembled with two kinds of achiral noncovalent frameworks (G1/CB[8] or G2/CB[8]) displayed different opposite circular dichroism signals based on different binding modes and affinity, achieving chirality transfer from tripeptide to organic supramolecular assemblies with further enhanced NIR fluorescence up to 46.2 times and the quantum yield (QY) increased from 0.71% to 10.29% for G2/CB[8], showing reversible chirality transfer and tunable NIR fluorescence under thermal stimulus. Therefore, the current research has achieved controllable chirality transfer from tripeptide to the SOFs and the enhancement of tunable NIR fluorescence, which is successfully applied in thermal responsive chiral logic gates, information encryption, and cell imaging.

## Graphical abstract

Chiral NIR supramolecular phenothiazine organic frameworks based on stepping confinement of macrocyclic and tripeptides assemble not only form opposite chiral noncovalent polymers and achieve thermally activation‐driven reversible chirality transfer, but also enhance the tunable NIR luminescence, which is successfully applied in chiral logic gates, information encryption, and cell imaging.

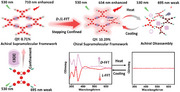

## 1 Introduction

Chiral supramolecular luminescent materials, especially those constructed by macrocyclic compounds including cyclodextrin,^[^ ^1^ ^]^ macrocyclic arenes,^[^ ^2^ ^]^ and cucurbit[n]uril (CB[n])^[^ ^3^ ^]^ to confine chromophore guests to induce or enhance the fluorescence or phosphorescence properties,^[^ ^4^ ^]^ and the circularly polarized luminescence,^[^ ^5^ ^]^ have become one of the research hotspots in supramolecular science, showing wide application in biological,^[^ ^6^ ^]^ information encryption,^[^ ^7^ ^]^ sensing,^[^ ^8^ ^]^ optical devices^[^ ^9^ ^]^ and catalysis.^[^ ^10^ ^]^ Generally, two strategies existed to realize the chiral luminescence of macrocyclic supramolecular materials. One is to use the rigid microenvironment of macrocycles to confine the guests, which can not only transfer the chirality of macrocycles to the luminophores but also enhance or activate the luminescence of the guests. For instance, Inouye et al.^[^ ^11^ ^]^ found that *γ*‐cyclodextrin included alkynylperylenes with terphenyl‐type stopper molecules, giving a [4]rotaxane with orange fluorescence, showing a high quantum yield of up to 15% and significant dissymmetry factor (*g_lum_* = −2.1 × 10^−2^). Liu and coworkers^[^ ^12^ ^]^ demonstrated that *β*‐cyclodextrin (*β*‐CD) could act as the chiral wheel to encapsulate aminopyrene derivative and dynamical condense with triformylphloroglucinol to form the chiral 2D covalent organic framework with tunable luminescence deriving from the confinement of *β*‐CD. Yang et al.^[^ ^13^ ^]^ synthesized a series of bipyrene‐modified cyclodextrin and found that one pyrene unit of the host could penetrate the cavity of another host to give a right‐handed excimer and further aggregate into nanobelt, and the quantum yield was enhanced to 64.0% in water. Nau et al.^[^ ^14^ ^]^ reported that 2,3‐diazabicyclo[2.2.2]oct‐2‐ene derivatives were encapsulated by *β*‐CD via parallel or perpendicular to the axis of the host to form co‐conformational host‐guest complexes, giving positive or negative CD signals respectively, and also provided an insight for the absolute conformation of the chiral supramolecular complexes. The other efficient strategy is utilizing chiral microenvironments between macrocyclic compounds and guests to form the chiral assembly and further doping with other achiral guests or dyes to achieve chirality transfer and luminescence. Duan and coworkers^[^ ^15^ ^]^ found that *β*‐CD confined sodium dodecyl sulfate to form the chiral host‐guest complex and assembled to give a chiral nanosheet, which could further dop with dyes such as thioflavin T or Nile red to form chiral luminescent materials. Although there have been some reports on the chirality of macrocyclic supramolecular assemblies induced by chiral molecules, however, taking advantage of the achiral macrocyclic host such as CB[n] to bind with guests to form NIR fluorescence supramolecular frameworks and then cascade assemble with chiral guest to induce thermally activation driven reversible chirality transfer, especially enhancing NIR fluorescence, is still rarely reported.

**Scheme 1:**
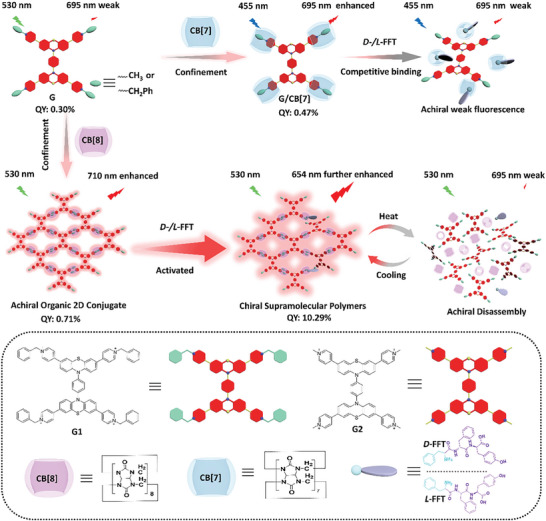
Configurationally stepping confinement achieved tunable chiral NIR luminescence supramolecular phenothiazine organic framework (partial CB[8] encapsulated tripeptide guests were shown in the coassembly for clarity).

In this work, we reported that the two kinds of paddle‐like phenothiazine derivatives containing four cationic terminal groups were encapsulated by CB[8] to form supramolecular organic frameworks not only enhanced NIR fluorescence emission with 25 nm hypsochromic or 15 nm bathochromic shift respectively, but also inducing the guest's morphology turned from irregular nanoparticles to organic nanosheets based on host‐guest complexation. Especially by the stepping assemble confinement, the supramolecular organic frameworks of G1/CB[8] or G2/CB[8] could further coassemble with tripeptides (*L*‐/*D*‐phenylalanyl‐*L*‐/*D*‐phenylalanyl‐*L*‐/*D*‐tyrosine, *L*‐/*D*‐FFT), giving opposite CD signals with different intensities at 550 nm by the same chiral guest, resulting in chirality transfer from tripeptide to 2D noncovalent frameworks, giving a 46.2 times enhancement of fluorescence intensity at 654 nm for G2/CB[8], showing thermal activation driven reversible chirality transfer and tunable NIR fluorescence emission. As can be seen from **Scheme** **1**, the configurationally stepping confined chiral NIR luminescent SOFs were constructed by the cascade macrocycle and secondary assembly confinement, which not only achieved the enhancement of NIR fluorescence and the transformation of assembly morphology to network SOFs but also enhanced the luminescence properties of supramolecules and also realized the reversible chirality transfer. Finally, the organic two‐dimensional supramolecular polymers were successfully used in chiral logic gates, information encryption, and NIR luminescence cell imaging, which also put forward a new insight for tunable chiral red luminescent supramolecular materials.

## 2 Results and Discussion

**Figure 1:**
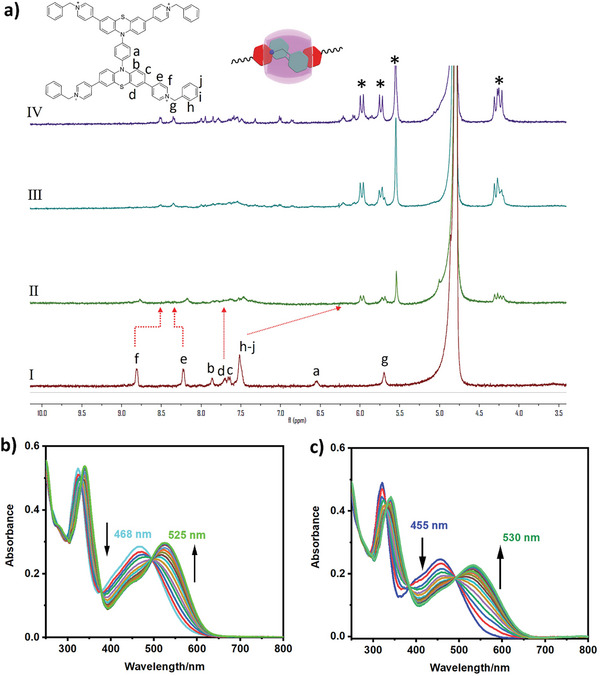
a) ^1^H NMR spectra of G1 with the addition of CB[8] ([G] = 0.02 mM, (I): 0 eq.; (II):0.5 eq.; (III):1.5 eq.; (IV): 2.0 eq. CB[8]; *: the protons of CB[8]) in D_2_O/(CD_3_)_2_SO = 95/5. UV–vis spectra of b) G1 and c) G2 with the addition of CB[8] (0–4.0 eq.) ([G1] = [G2] = 0.01 mM).

First, two kinds of tetracationic bis(phenothiazines) were synthesized by bromination, coupling, and quaternization reaction according to the designed synthesize route (Scheme S1, Supporting Information). The structures of the two guests were investigated by nuclear magnetic resonance (NMR) and high‐resolution mass spectrometry (Figures S1–S8, Supporting Information). The ^1^H NMR titration experiments (**Figure** **1**a) showed that the protons of benzyl (H_h‐j_) and pyridiniums (H_f_) in G1 were upfield shifted due to the shielding effect of the host, indicating the benzyl and partial pyridyl (H_f_) units were included in CB[8]’s cavity.^[^ ^16^ ^]^ However, the proton of the pyridinium (H_e_) was down‐field shifted, suggesting the partial structure of the cationic group was on the CB[8] portal,^[^ ^17^ ^]^ thus positively charged benzyl unit of G1 should be deeply included by the cavity of macrocyclic host. Furthermore, the pyridinium containing the benzyl group (G3) was selected as the model compound and ^1^H NMR titration experiments were performed. Figure S9 (Supporting Information) showed that the protons of the benzene (H_a‐c_), methylene group (H_d_), and the pyridinium (H_e_) gave upfield shift, while the residual protons of pyridiniums (H_f‐g_) were down‐field shifted in the presence of CB[8], implying the benzene group was included by CB[8]. Additionally, the 2D NOESY NMR spectrum of G1/CB[8] showed that the benzyl and partial pyridyl (H_f_) units were included in CB[8]’s cavity (Figure S10, Supporting Information) confirming the simulation mode of G1/CB[8] (Figure S11, Supporting Information). In sharp contrast with G1, the protons of pyridinium (H_e‐f_) and methyl group (H_g_) in G2 (Figures S12 and S13, Supporting Information) exhibited apparent upfield shift and gradually broadened under the same conditions, manifesting the pyridiniums were deeply encapsulated by CB[8] and may further lead to the formation of periodic supramolecular noncovalent polymers.^[^ ^18^ ^]^ Compared with CB[8], possessing a smaller hydrophobic cavity, CB[7] can only bind with one guest.^[^ ^19^ ^]^ The binding mode between the guest molecules and CB[7] was also studied. In Figures S14 and S15 (Supporting Information), the protons of the benzyl group in G1 and the pyridinium unit of G2 were upfield shifted in the presence of CB[7], indicating the CB[7] encapsulated the benzyl group of G1 and the pyridinium unit of G2, respectively. Furthermore, the 2D NOESY NMR experiments further confirmed the cationic arms of the guests were encapsulated by the CB[7] and formed host‐guest complexes (Figures S16 and S17, Supporting Information).

To further investigate the binding behaviors of cucurbituril to cationic bis(phenothiazines) derivatives, UV–vis spectroscopy experiments were performed to determine the binding constants and stoichiometric ratio. As for G1, the characteristic absorption signal at 468 nm gradually decreased, and the peak at 525 nm increased under the confinement of CB[8] (Figure 1b). The G2 gave a larger redshift up to 75 nm after complexing with the host (Figure 1c), which may be ascribed to the pyridinium groups of G2 were included by CB[8] in the “head‐to‐tail” pattern and forming *J* aggregates,^[^ ^20^ ^]^ thus leading to a larger redshift in the absorption spectrum. In Job's plots, the inflection points were located at 0.33 (Figure S18, Supporting Information), indicating the stoichiometric ratio between the tetracationic guests with different terminal groups and CB[8] was 1:2 in the assembly of G1_n_/CB[8]_2n_ and G2_n_/CB[8]_2n_ (n was the number of repeated units). The binding constant (*K_s_*) between G1 and CB[8] was calculated to be 1.30 × 10^12^ m ^−2^ (Figure S19, Supporting Information) by the change of UV–vis spectra at 525 nm according to the nonlinear least‐squares fitting formula.^[^ ^21^ ^]^ Compared with G1, the supramolecular complex of G2 with methyl pyridinium terminal groups and CB[8] gave smaller binding constants (Figure S20, Supporting Information, 7.19 × 10^10^ m ^−2^), which may be ascribed to the different binding sites between the two kinds of guests and CB[8] that was the benzyl pyridinium units of G1 could be more deeply and tightly encapsulated by CB[8]. The above result of experiments showed that both of the two kinds of cationic bis(phenothiazines) derivatives were included by CB[8] in the same stoichiometric ratio and showed strong binding constants; thus, G1 or G2 could complex with CB[8] by strong noncovalent interaction to give stable host‐guest complexes and then assembled to form more extended supramolecular conjugates. As for two kinds of phenothiazine derivatives and CB[7], the inflection point in Job's plot was 0.2 (Figure S21, Supporting Information), manifesting the stoichiometric ratio between guests and CB[7] was 1:4. In Figure S22 (Supporting Information), the apparent binding constants of CB[7] to G1 or G2 were also studied and measured as 3.52 × 10^4^ m ^−1^ and 4.26 × 10^4^ m ^−1^, respectively, giving weak binding affinity than the complexes of G1/CB[8] or G2/CB[8], and maybe since two kinds of guests bind with CB[7] could only form simple host‐guest complexes.

**Figure 2:**
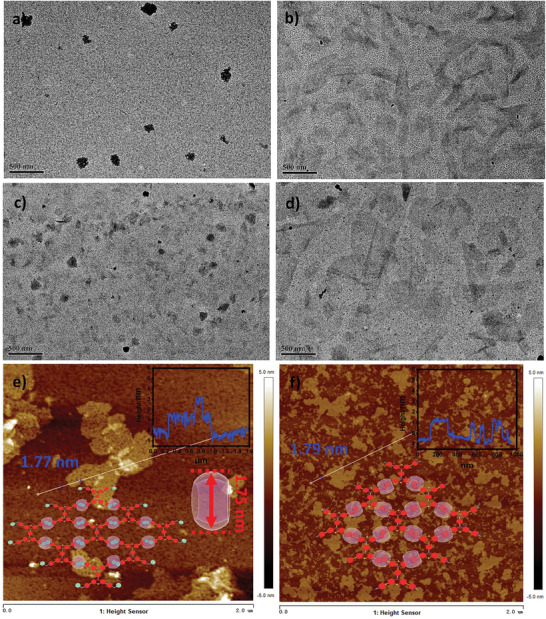
TEM images of a) G1 and b) G1/CB[8]. TEM images of c) G2 and d) G2/CB[8]. AFM image of e) G1/CB[8] and f) G2/CB[8] (inset: height section profiles and schematic illustration of the assembled models of G1/CB[8] and G2/CB[8], respectively).

The assembly behaviors and topological morphology of the supramolecular assemblies were studied by dynamic light scattering (DLS), zeta potential experiments, transmission electron microscopy (TEM), and atomic force microscopy (AFM). DLS results manifesting both G1/CB[8] and G1/CB[8] gave large hydrodynamic sizes, respectively (Figure S23a, Supporting Information), which excludes the formation of simple host‐guest complexes. The Zeta potentials of the two assemblies were 21.3 and 4.02 mV (Figure S23b, Supporting Information), respectively, indicating that the surface of noncovalent polymers was positively charged. In TEM images, compared with the irregular nanoparticles formed by G1 (**Figure** **2**a), the assembly morphology of G1/CB[8] was turned into nanoflakes (Figure 2b) under the confinement of CB[8]; the lamellar sheet was also observed in AFM picture (Figure 2e), and the thickness of the nanosheets was measured to be 1.77 nm, which was identical to the outer diameter of CB[8] (1.75 nm).^[^ ^22^ ^]^ G2 also gave a similar assembled morphology by the confinement of CB[8] (Figures 2d,f). Furthermore, AFM images of G1/CB[8] and G2/CB[8] prepared by the spin‐coating method further confirmed the nanosheet‐like structures of the assembly in solution (Figure S24, Supporting Information). In addition, both G1/CB[7] and G2/CB[7] gave irregular nanostructures (Figure S25, Supporting Information), indicating these simple host‐guest complexes could not assemble to form ordered supramolecular assemblies. Therefore, benefiting from CB[8] has a large cavity that can include two cationic guests, the positively charged paddles in two kinds of tetracationic bis(phenothiazines) can stack in a “head‐to‐tail” mode, leading to firm complexes of G1/CB[8] or G2/CB[8], and further assemble to large extended lamellar sheet SOFs (Figures inset 2e,f).

**Figure 3:**
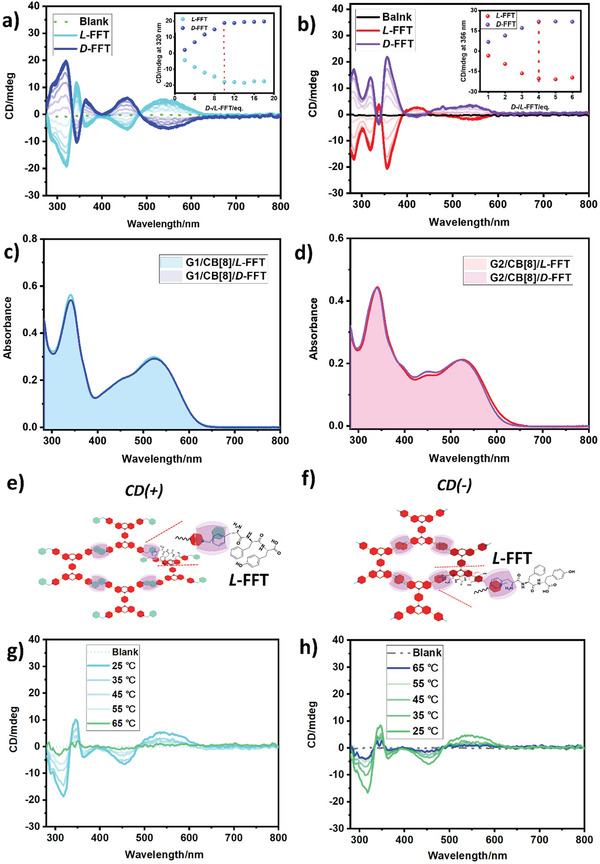
a) The CD spectra of G1/CB[8] with the addition of *D*‐/*L*‐FFT. Inset: the CD signal of G1/CB[8] at 320 nm in the presence of *D*‐/*L*‐FFT (0–18.0 eq.). b) The CD spectra of G2/CB[8] with the addition of *D*‐/*L*‐FFT. Inset: the CD signal of G2/CB[8] at 356 nm in the presence of *D*‐/*L*‐FFT (0‐6.0 eq.) ([G1] = [G2] = 0.05 mM, [CB[8]] = 0.1 mm). c) UV–vis spectra of G1/CB[8] with *D*‐/*L*‐FFT (10.0 eq.). d) UV–vis spectra of G2/CB[8] with with *D*‐/*L*‐FFT (4.0 eq.) ([G1] = [G2] = 0.01 mM, [CB[8]] = 0.02 mM). Schematic illustration of the adaptive chirality of e) G1/CB[8]/*L*‐FFT and f) G2/CB[8]/*L*‐FFT. The CD spectra of G1/CB[8]/*L*‐FFT g) heated and h) cooled at different temperatures.

The fabricated SOFs based on CB[8] confinement possess paddle‐shaped phenothiazine units and macrocyclic cavities, which may further coassemble with chiral molecules by multivalent interaction, including host‐guest and electrostatic interaction to form functional supramolecular assemblies. Therefore, can we utilize the advantage of CB[8] binding with *D*‐/*L*‐phenylalanine to induce achiral assembly to generate chirality? To this end, a series of chiral phenylalanine derivatives (Figure S26, Supporting Information) were selected to coassemble with achiral noncovalent polymers and further investigated the chirality transfer from guests to the assemblies. In Figure S27a,b (Supporting Information), no induced CD signal was observed in the absorption band of two kinds of SOFs (300–650 nm) in the presence of chiral molecules such as *L*‐phenylalanine (*L*‐Phe), *L*‐phenylalanyl‐*L*‐phenylalanine (*L*‐FF), Cefalexin and Nateglinide. The achiral G1/CB[8] was induced to give the CD signal at 300–650 nm (**Figure** **3**a) with the increase of tripeptide concentration and reached the maximum at 10.0 eq. (Figure 3a, inset), and corresponding to the UV–vis spectrum of assemblies (Figure 3c); the mirror‐symmetric CD spectrum was also observed after the addition of *D*‐FFT, indicating the chirality was transferred from the tripeptide to the assemblies, showing adaptive chirality signals. The positive or negative CD signals of chiral supramolecular assembly at the maximum wavelength corresponded to the *M* or *P* rotation conformation of phenothiazine derivatives in noncovalent polymers, respectively.^[^ ^23^ ^]^ Compared with G1/CB[8], the coassembly of G2/CB[8]/*L*‐FFT exhibited the negative CD peak at≈550 nm with different intensities (Figure 3b) and reached equilibrium at 4.0 eq. of tripeptide (Figure 3b, inset), and corresponding to the UV–vis absorption peaks of G2/CB[8]/tripeptides (Figure 3d), indicating the G2/CB[8] generated the opposite adaptive chirality to G1/CB[8] under the same conditions. This interesting result attracted us to investigate the influence of the coassemble behavior on the chirality transfer from tripeptide to achiral SOFs. As shown in Figure S28 (Supporting Information), with the addition of *L*‐FFT, the proton signals of CB[8] at 5.5–6.0 ppm in G1/CB[8] were broadened, and the signal of benzene unit at 6.83 ppm in tripeptide was upfield shifted, indicating *L*‐FFT was included by CB[8] and formed heterodimers with the cationic arms of G1. Additionally, in G3/CB[8] (Figure S29, Supporting Information), the protons of methylene in *L*‐FFT were downfield shifted at 3.18 ppm, indicating the methylene connected to the benzene at the terminal group of tripeptide was located on the portal of CB[8]; thus, we reasonably inferred that the *L*‐FFT was shallowly included by CB[8] in the assembly of G1/CB[8] (Figure S30, Supporting Information). It's worth noting that the methylene proton of tripeptide was upfield shifted from 3.05 to 2.52 ppm in G2/CB[8]/*L*‐FFT (Figure S31, Supporting Information), which should be attributed to the benzene unit of *L*‐FFT and methylene unit were deeply encapsulated by CB[8] in the coassembly. Therefore, the two kinds of SOFs could assemble with the same tripeptide at different binding modes to form multivalent assemblies. On the other hand, the binding affinity of noncovalent frameworks to *L*‐FFT was also investigated by UV‐vis spectroscopy titration experiments. As shown in Figure S32 (Supporting Information), the G2/CB[8] and *L*‐FFT gave more strong apparent binding constants (3.45 × 10^4^ M^−1^) than that G1/CB[8] with tripeptide (2.37 × 10^3^ M^−1^), which may ascribe to the stronger affinity of CB[8] to G1 than G2. Thus, as the competitive guest, the *L*‐FFT is easier to co‐assemble with the guests with methyl pyridinium arms (G2) to form the chiral assembly. More obviously, with the addition of tripeptide, the absorption peak of G2/CB[8] at 455 nm gradually increased (Figure S32c, Supporting Information), corresponding to the absorption peak of the guest, implying the formation of heterodimers between *L*‐FFT and G2 under the confinement of CB[8] and disturbed the *J* aggregates of G2. Although both the two assemblies showed similar lamellar nanostructures in TEM images (Figure S33, Supporting Information), G2/CB[8]/*L*‐FFT gave a smaller assembled size than G1/CB[8]/*L*‐FFT in TEM and DLS experiments (Figure S34a, Supporting Information), which may be ascribed to the stronger affinity between G2/CB[8] and *L*‐FFT was more conducive to the formation of stable chiral assembly. The different binding modes and behaviors between tripeptide and two kinds of achiral SOFs may induce conformational changes in the tripeptide, and further lead to different CD signals derived from chiral noncovalent polymers. The *L*‐tripeptide gave a positive CD signal at 226 nm (Figure S35, Supporting Information), which implied the *β*‐turn‐like fold conformation of *L*‐FFT existed in the solution.^[^ ^24^ ^]^ The positive Cotton effect was observed at≈550 nm in the chiral assembly of G1/CB[8]/*L*‐FFT, suggesting the chirality of the *L*‐tripeptide transferred to the coassembly with the *M*‐conformation, which may be due to the folded conformational *L*‐FFT was the shallow encapsulated by CB[8] in the chiral noncovalent framework (Figure 3e). Compared with G1, G2 with methyl pyridinium terminal groups gave weaker binding ability with CB[8] and was more conducive to the deep encapsulation of phenylalanine terminal groups of *L*‐FFT by CB[8], leading to *L*‐FFT transform into unfolded conformation (Figure 3f), and then inducing achiral SOF to give *P*‐conformational chirality, thus showing negative CD signal from G2/CB[8]/*L*‐FFT. Therefore, the CB[8] confined two kinds of guests with different cationic terminal units to form achiral supramolecules and further cascade assembled with the same chiral guest to give different opposite CD signals and adaptive chirality. Furthermore, the supramolecular complexes of CB[7] and two kinds of guests cannot be induced by *L*‐FFT to give the CD signal in the UV–vis absorption region of the tetracationic bis(phenothiazines) (Figure S36a,b, Supporting Information), which further confirms that the confinement effect of CB[8] can bind two different guest molecules is indispensable for chiral transfer. The macrocyclic supramolecules based on host‐guest interaction possess dynamic reversibility, which may endow the chiral assemblies with thermal responsive properties. Figure 3g showed that the CD signal of G1/CB[8]/*L*‐FFT at 550 nm gradually disappeared when heated to 65 °C, indicating the chirality transfer from tripeptide to the assemblies was interrupted, which should ascribe to the disassemble of the chiral assemblies. Then, the chiral signal of the supramolecular polymer was gradually recovered after cooling at 25 °C (Figure 3h), thus the CB[8] confined chiral supramolecular assembly exhibited reversible chirality transfer under the thermal stimulus. Similarly, the G2/CB[8]/*L*‐FFT also exhibited reversible thermal responsive chirality transfer properties (Figure S36c,d, Supporting Information).

**Figure 4:**
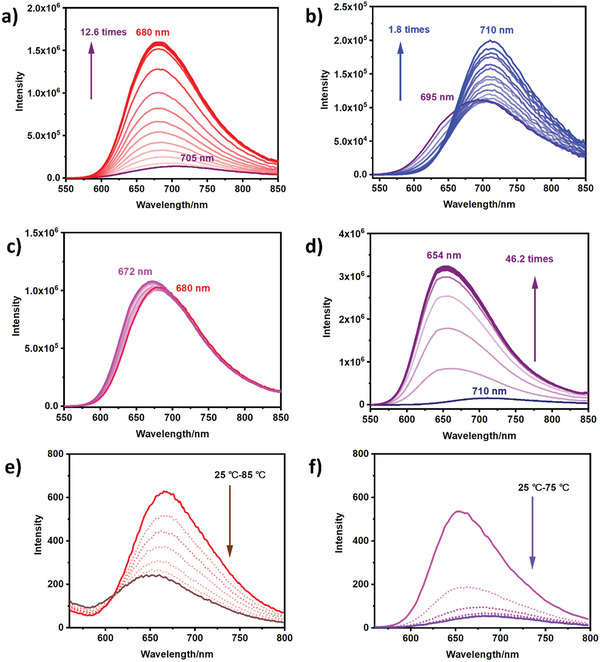
a) Fluorescence spectra of G1 with the addition of CB[8] (0–3.0 eq.) (λ_ex_ = 525 nm). b) Fluorescence spectrum of G2 with the addition of CB[8] (0–3.0 eq.) (λ_ex_ = 530 nm, [G1] = [G2] = 0.01 mM). c) Fluorescence spectrum of G1/CB[8] with the addition of *L*‐FFT (0‐10.0 eq.) (λ_ex_ = 525 nm). d) Fluorescence spectrum of G2/CB[8] with the addition of *L*‐FFT (0‐10.0 eq.) (λ_ex_ = 530 nm, [G1] = [G2] = 0.01 mM, [CB[8]] = 0.02 mM). Fluorescence spectra of (e) G1/CB[8]/*L*‐FFT (λ_ex_ = 525 nm, [G1] = 0.01 mM, [CB[8]] = 0.02 mM, [*L*‐FFT] = 0.1 mM) and (f) G2/CB[8]/*L*‐FFT (λ_ex_ = 530 nm, [G2] = 0.01 mM, [CB[8]] = 0.02 mM, [*L*‐FFT] = 0.04 mM) heated at different temperatures.

Significantly, CB[8] can not only confine the tetracationic phenothiazine derivatives to form network supramolecular frameworks through the host‐guest interaction but also improve its NIR fluorescence properties. **Figure** **4**a showed that G1 gave a weak fluorescence signal at 705 nm, which further blue‐shifted to 680 nm along with the increasing CB[8] concentration and the fluorescence intensity was enhanced 12.6 times. Unlike G1, G2 with methyl terminal unit gave a 25 nm redshift and just enhanced 1.8 times at 710 nm in the presence of CB[8] (Figure 4b), which may be due to the *J* aggregates formed by G2 with methyl terminal units under the confinement of CB[8]. We can reasonably speculate that the larger binding affinity of CB[8] to G1 is beneficial to form a more stable supramolecular conjugate and can effectively restrict the movement of excited phenothiazine luminophores, leading to more efficient fluorescence emission. The confinement effect of CB[7] for the two kinds of guests was also studied. In Figure S37 (Supporting Information), the fluorescence peak of G1 blueshifted to 640 nm, and the luminescence intensity was enhanced 14.6 times. However, the NIR fluorescence intensity of G2 gave a 1.3 times enhancement, which may be ascribed to the different binding behaviors between two kinds of guests and CB[7]. Additionally, the QY of G1 (Figure S38, Supporting Information) was increased from 0.65% to 5.81%, and 6.32% under the confinement of CB[8] and CB[7]. The system of G2/CB[8] and G2/CB[7] just gave slightly enhanced QY from 0.3% (G2) to 0.71% and 0.47% respectively (Figure S39, Supporting Information), which further confirmed CB[n] confinement was beneficial to the luminescence of guests.

Notably, the stepping assemble confinement of SOFs and tripeptide can realize chirality transfer and improve the NIR luminescence of chiral noncovalent polymers. As can be seen from Figure 4c, the G1/CB[8] conjugate exhibited an 8 nm blueshift after *L*‐FFT was added, and there was no obvious change in luminescence intensity. However, the fluorescence intensity of the G2/CB[8] was enhanced up to 46.2 times, and the NIR emission peak was blueshifted to 654 nm in the presence of *L*‐FFT (Figure 4d). In addition, the QY of G2/CB[8] was increased from 0.47% to 10.29% after coassembling with the tripeptide (Figure S40, Supporting Information), while the QY of G1/CB[8] was almost unchanged (from 5.81% to 5.87%). In sharp contrast with G2/CB[8], the fluorescence intensity of G1/CB[7] and G2/CB[7] were greatly decreased in the presence of tripeptide (Figure S41a,b, Supporting Information) and the QY was also reduced to 2.28% and 0.37% (Figure S41c,d, Supporting Information) respectively, which may be ascribed to the competitive binding of *L*‐FFT to the CB[7]. The following reasons may explain this interesting result that is the stronger binding constant between *L*‐FFT and G2/CB[8] is beneficial to form stable heterodimers, restricting the movement of luminophores, then is convenient to the formation of the stable coassembly and reducing the collision of solvent molecules, leading to effectively enhanced fluorescence emission. Additionally, the fluorescence properties of the two kinds of coassemblies also exhibited thermal responsiveness. As shown in Figure 4e, the fluorescence intensity of G1/CB[8]/*L*‐FFT was gradually decreased with the temperature increasing and the fluorescence quenching efficiency was calculated to be 63.11% (85 °C). The NIR fluorescence of the coassembly was recovered to some extent when cooled to 25 °C (Figure S42a,b, Supporting Information). It is worth noting that the G2/CB[8]/*L*‐FFT is more sensitive to temperature changes, giving a fluorescence quenching efficiency of up to 92.01% (Figure 4f), which may be caused by the disassemble of the G2/CB[8]/*L*‐FFT under thermal stimulation.

**Figure 5:**
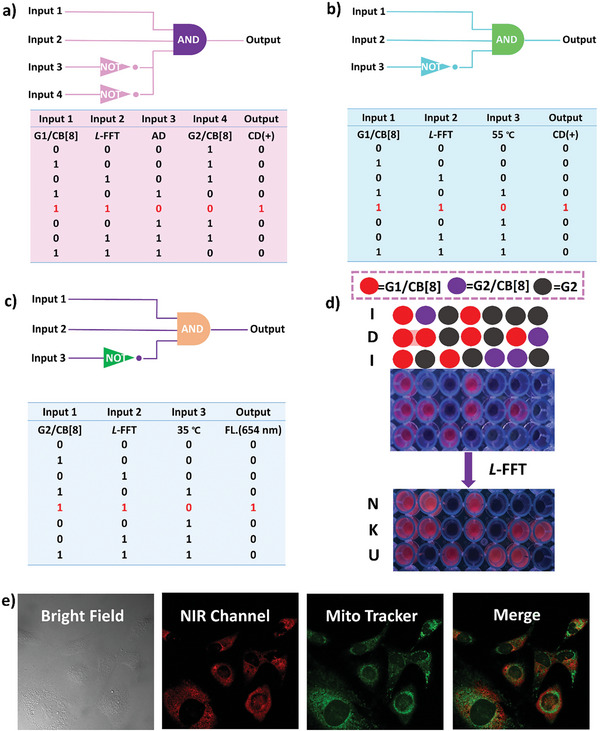
Scheme representation for a) competitive guest, thermal‐regulated INHIBIT b) chiral and c) fluorescence logic gates. d) Schematic illustration of information encryption: the Morse code under 365 nm light. e) Confocal microscopy images of HeLa cells in the presence of G2/CB[8]/L‐FFT and Mito Tracker green ([G2] = 0.01 mM, [CB[8]] = 0.02 mM, [*L*‐FFT] = 0.04 mM).

Considering that the fabricated chiral supramolecular assemblies possess excellent NIR luminescent properties and adjustable chirality, we explored the possible application of the assemblies in chiral logic gates, information encryption, and cell imaging. In **Figure** **5**a, the CD and NIR fluorescence signals were defined as “output”, and tripeptide, SOFs, and other controlling conditions were defined as “input”. The CD signal intensity at 550 nm exceeds 5 or the fluorescence intensity at 654 nm more than 200 (Figure 4f) was defined as “1”, and below that was set as “0”. As shown in Figure S43 (Supporting Information), after the addition of cationic adamantane (AD), the CD signal of chiral assembly at 550 nm was decreased and disappeared, which was due to the strong bind interaction between AD and CB[8],^[^ ^25^ ^]^ resulting in the disappearance of chirality transfer. Therefore, cationic adamantane was set as “not gate”, and the CD signals could not observed in the coexistence of the tripeptide, SOF, and AD. In addition, the achiral G2/CB[8] can also be set as the “NOT gate” due to which coassembles with *L*‐FFT to give a negative CD signal and cooperates with competitive guest AD to develop as a multi‐responsive logic gate, then outputting more reliable information. Furthermore, the chiral supramolecular system exhibited thermal‐responsive reversible chirality transfer and NIR luminescence, thus, the temperature condition was set as the “NOT gate”, and the CD and NIR fluorescence signals could not output (Figure 5b,c). This multi‐component chiral supramolecular logic gate features multi‐stimulus responsiveness and controllability, which can output complex information and be used as a reliable logic gate system. Additionally, the red fluorescence supramolecular assemblies based on the confinement of CB[8] were also applied in information encryption. Figure 5d showed that G1/CB[8], G2/CB[8], and G2 alone were added to the corresponding position of the 96‐well plate, and the invalid information “IDI” was read out by the translated morse code under 365 nm light, however, the accurate information “NKU” was decoded after the addition of tripeptide.

The chiral supramolecular assembly possesses good NIR luminescence properties, and cascade assembles to form nanosheets, which may be used as luminescent materials for cell imaging. G2/CB[8] and G2/CB[8]/*L*‐FFT were incubated with HeLa cells for 24 h respectively, and analyzed by confocal laser scanning microscopy. Different from the very weak red signal of G2/CB[8] in Figure S44 (Supporting Information), the chiral coassembly exhibits bright red fluorescence (Figure 5e, NIR channel), which may be due to the better fluorescence properties of chiral coassembly possessing the smaller assemble size is convenient to entry cells by endocytosis,^[^ ^26^ ^]^ and giving bright red luminescence in cells. Furthermore, the red chiral coassembly was added to the HeLa cells and treated with nuclei dye Hoechst (Figure S45, Supporting Information) and Mito tracker (mitochondrial targeting reagent), respectively, showing well‐overlapped sites with the Mito‐tracker reagent (Figure 5e, Merge), indicating the chiral assembly could selectively label mitochondria.

## 3 Conclusion

In conclusion, taking advantage of stepping confinements of macrocyclic and assemble, we successfully fabricated the chiral guest‐inducing achiral organic frameworks to form thermal responsive chiral supramolecules by chirality transfer and enhancing the NIR luminescence of the chiral supramolecular assemblies. Different from the weak luminescence phenothiazine derivatives, CB[8] confined G1 or G2 not only induced the tetracationic guests to form network‐like noncovalent frameworks but also enhanced NIR fluorescence emission, which could cascade assemble with *L*‐/*D*‐tripeptide to give mirror‐symmetric circular dichroism signal and further enhanced 46.2 times NIR fluorescence with QY up to 10.29%. Benefiting from the different binding behaviors in the assemblies, two kinds of noncovalent frameworks were induced to generate positive or negative circular dichroism peaks with different intensities after coassembled with the same tripeptide, achieving guest chirality transfer to the achiral supramolecular frameworks, realizing the reversible chirality transfer and tunable NIR fluorescence under the activation of thermal. The present work is not only conducive to further understanding the mechanism of stimulus‐responsive chirality transfer from single molecule to achiral luminescent assembly but also successfully applied in chiral NIR luminescence logic gates, information encryption, and cell imaging.

## Conflict of Interest

The authors declare no conflict of interest.

## Supporting information



Supporting Information

## Data Availability

The data that support the findings of this study are available from the corresponding author upon reasonable request.
