# Supplementary material for: Configurationally Stepping Confinement Achieved Tunable Chiral Near‐Infrared Luminescence Supramolecular Phenothiazine Organic Framework
Source: Adv Sci (Weinh). 2024 Sep 20;11(42):2408107. doi: 10.1002/advs.202408107 (PMC11558156; doi:10.1002/advs.202408107)
Supplement: Supplementary file 1 — Supporting Information [file ADVS-11-2408107-s001.docx]

**Supporting Information**

**Configurationally Stepping Confinement Achieved Tunable Chiral Near-Infrared Luminescence Supramolecular Phenothiazine Organic Framework**

Jie Yu, Jie Niu, Xiufang Xu, and Yu Liu*

College of Chemistry, State Key Laboratory of Elemento-Organic Chemistry, Nankai University Tianjin 300071, P. R. China

E-mail: yuliu@nankai.edu.cn

**Materials:** The chemicals and solvents were purchased from HEOWNS; the *D*-/*L*-FFT was purchased from Nanjing Peptide Biotech Ltd. Silica gel (200-300 mush) and thin layer chromatography (TLC, GF254) were used for chromatographic purification and monitor the reaction, respectively. HeLa cells were purchased from the Cell Resource Center, China Academy of Medical Science Beijing, China,

**Measurements.** ^1^H NMR (400 MHz) and ^13^C NMR (100 MHz) spectra were performed on the Bruker Avance spectrometers, chemical shifts (δ) were labeled as ppm, and TMS was applied as the internal standard in NMR experiments. The fluorescence spectra were performed on the Edinburgh Instrument FS5. The TEM pictures were collected from the high-resolution transmission electron microscope (Philips Tecnai G2 20 S-TWIN microscope). Bruker Dimension Ion AMF was applied to capture the AFM images. Leika S8 microscope and Olympus FV1000 Laser scanning confocal microscope were applied to collect the cell images. The CD spectra were performed on the BioLogic MOS-500.

1. **The Synthesis route of cationic phenothiazine derivatives (Scheme S1)**


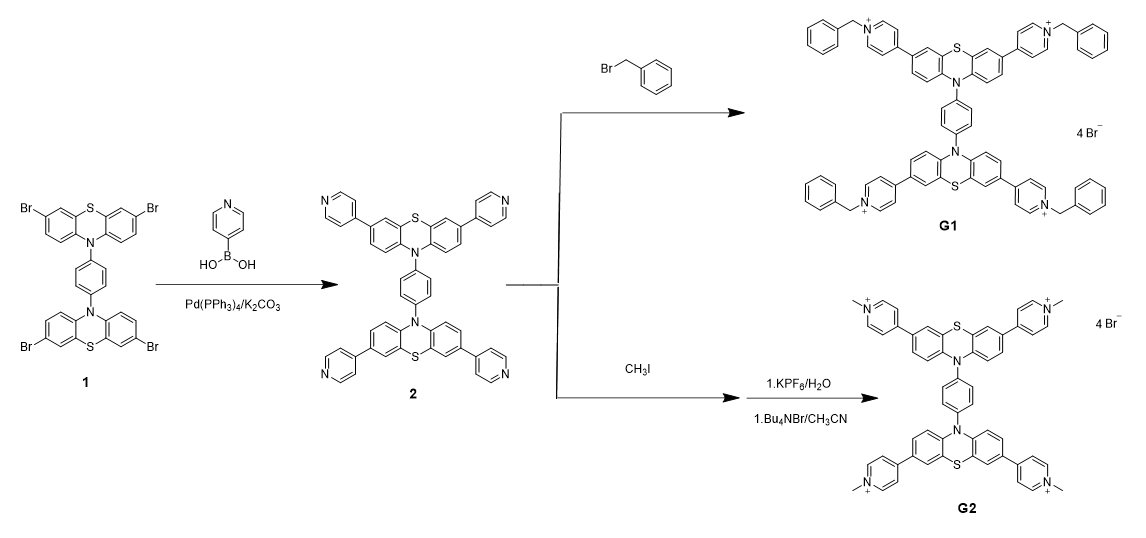


- 1. **The Synthesis of 2**


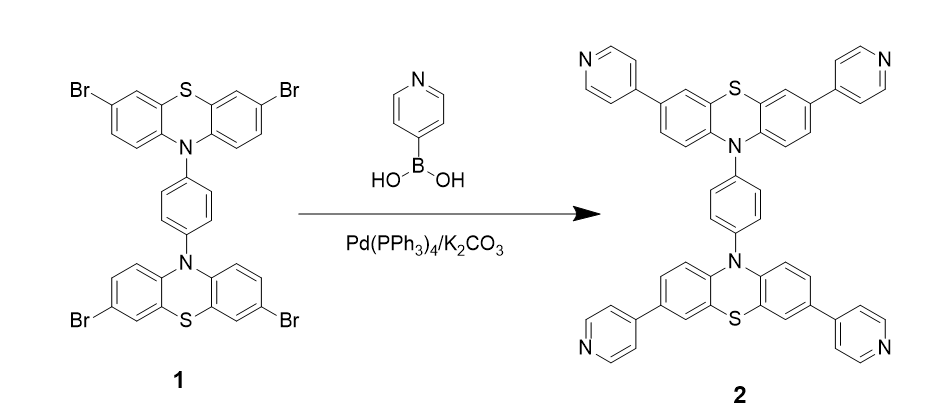


Pyridyl-4-boronic acid (0.94 g, 7.61 mmol) and compound 1 (1.00 g, 1.27 mmol) were dissolved in THF, then the solution of K_2_CO_3_ (2 M, 10 mL) was injected and bubbled with N_2_ for 30 min, after that the Pd (PPh_3_)_4_ (0.15 g, 0.13 mmol) were added in the solution. The reaction mixture was heated at 85 ℃ for 72 h, added to the water, and extracted with dichloromethane. The obtained dichloromethane layer was washed with water, saturated sodium chloride solution, and dried by anhydrous sodium sulfate, then evaporated under vacuum and purified by column chromatography to give compound 2 (0.34 g, yield: 34.3%). ^1^H NMR (400 MHz, (CD_3_)_2_SO): δ 8.60 (d, *J* = 5.9 Hz, 8H), 7.84 (s, 4H), 7.67 (m, 12H), 7.53 (m, 4H), 6.47 (d, *J* = 8.5 Hz, 4H). HR-MS (ESI): m/z for C_50_H_32_N_6_S_2_ calcd. [M+H]^+^= 781.2130, found: 781.2205.


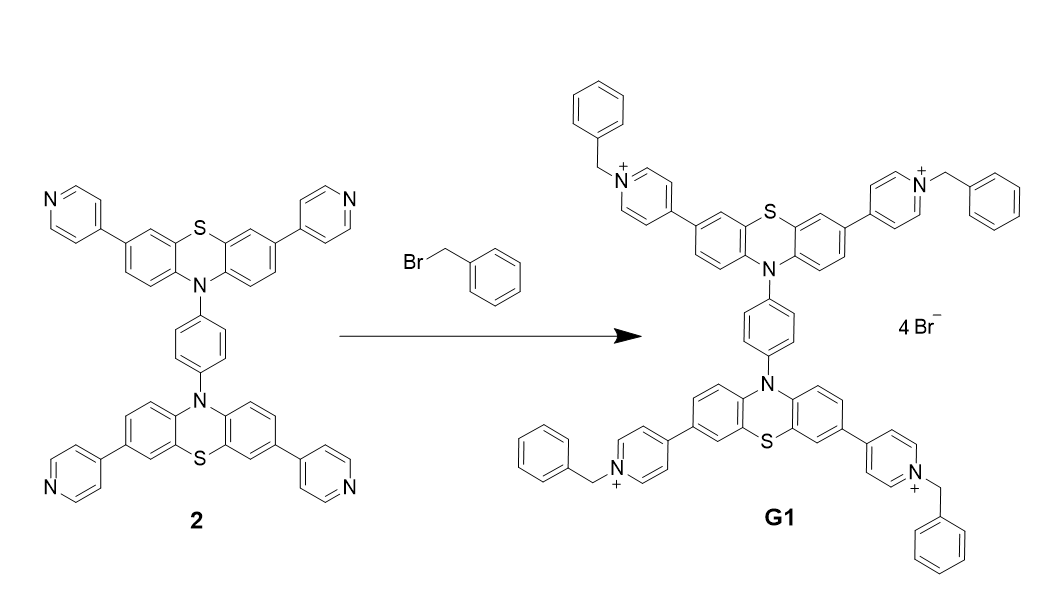


Compound 2 (100 mg, 0.13 mmol) was dissolved in dry DMSO, and then benzyl bromide (131 mg, 0.77 mmol) was injected into the solution and further heated at 80 ℃ for 24 h. The above reaction mixture was poured in diethyl ether to obtain the precipitate, which was further washed by diethyl ether and dried under vacuum to give dark solid G1 (65 mg, yield 34.6 %). ^1^H NMR (400 MHz, (CD_3_)_2_SO): δ 9.17 (d, *J* = 6.5 Hz, 8H), 8.46 (d, *J* = 6.7 Hz, 8H), 7.95 (d, *J* = 5.8 Hz, 8H), 7.78 (d, *J* = 6.6 Hz, 4H), 7.52 (m, 20 H), 6.43 (d, *J* = 8.8 Hz, 4H), 5.80 (s, 8H). ^13^C NMR (100 MHz, (CD_3_)_2_SO): δ 153.38, 145.50, 144.98, 140.06, 135.05, 134.34, 129.80, 129.72, 129.18, 128.72, 128.58, 126.81, 123.97, 120.20, 117.30, 62.70. HR-MS (ESI): m/z for C_78_H_60_N_6_S_2_Br_4_ calcd. [M-4Br]^4+^= 286.1075, found: 286.1089.

**
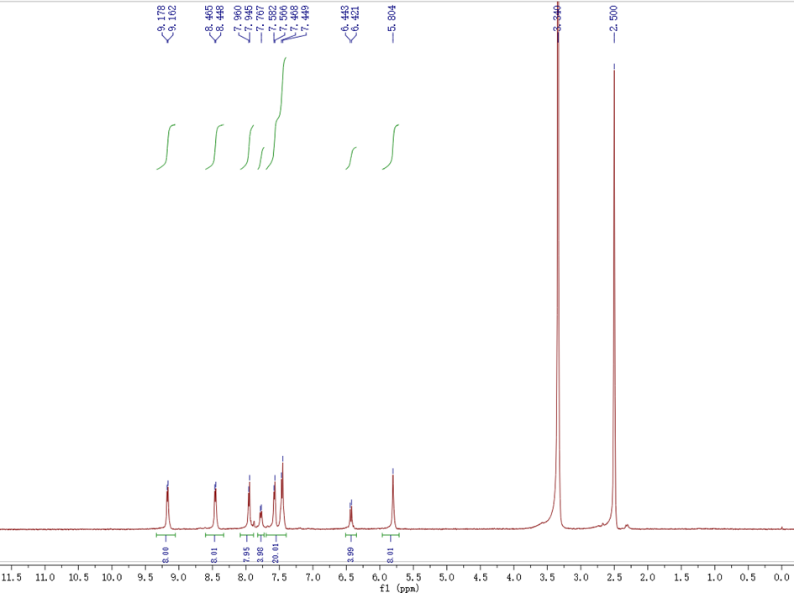
**

**Figure S1**. ^1^H NMR (400 MHz) spectrum of G1 in (CD_3_)_2_SO at 25 ℃.


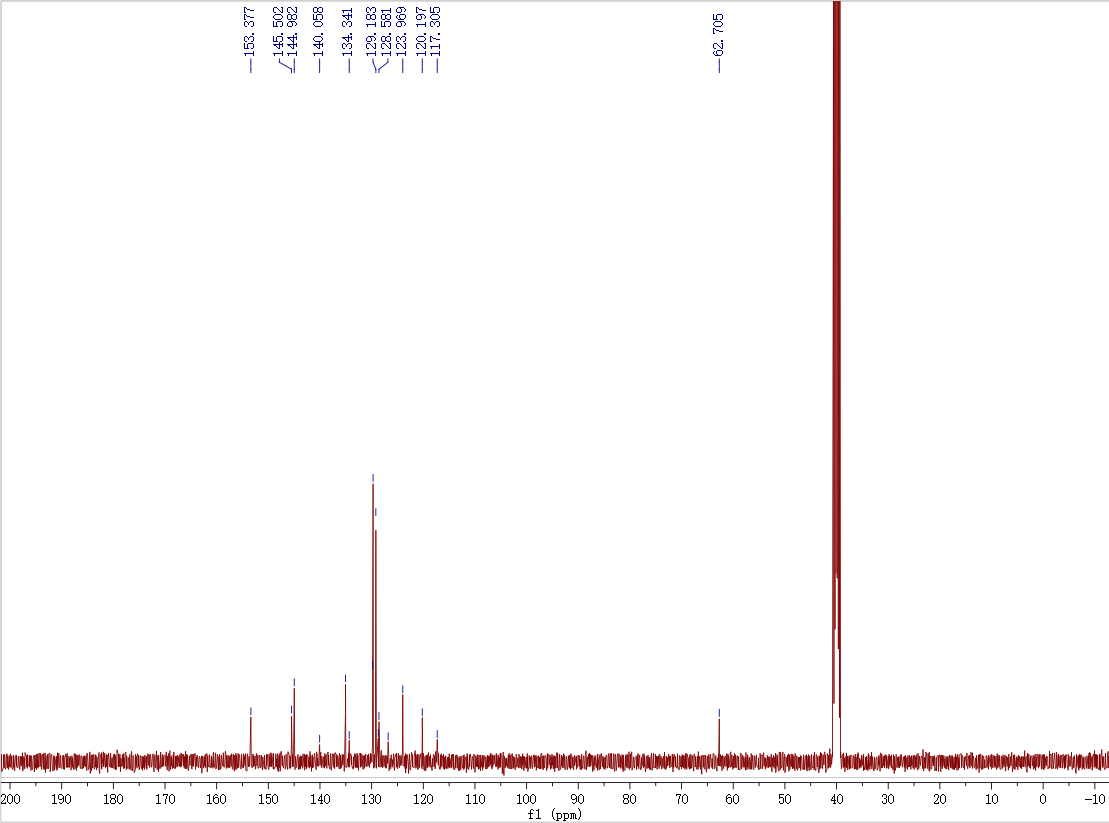


**Figure S2.** ^13^C NMR (100 MHz) spectrum of G1 in (CD_3_)_2_SO at 25 ℃.


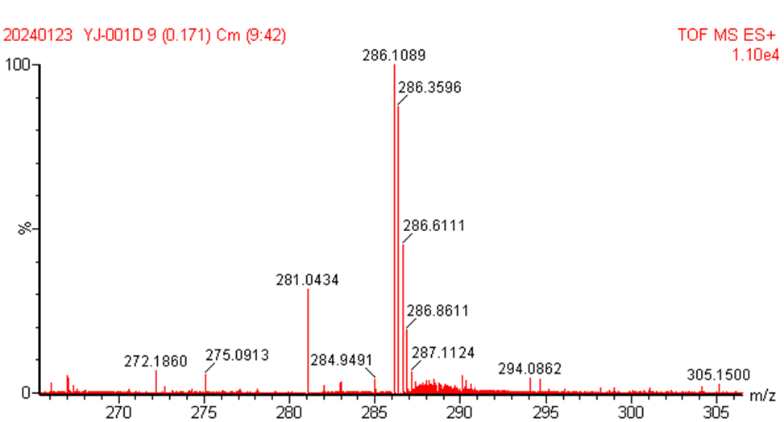


**Figure S3.** HR-MS spectrum of G1.

- 1. **The Synthesis of G2**


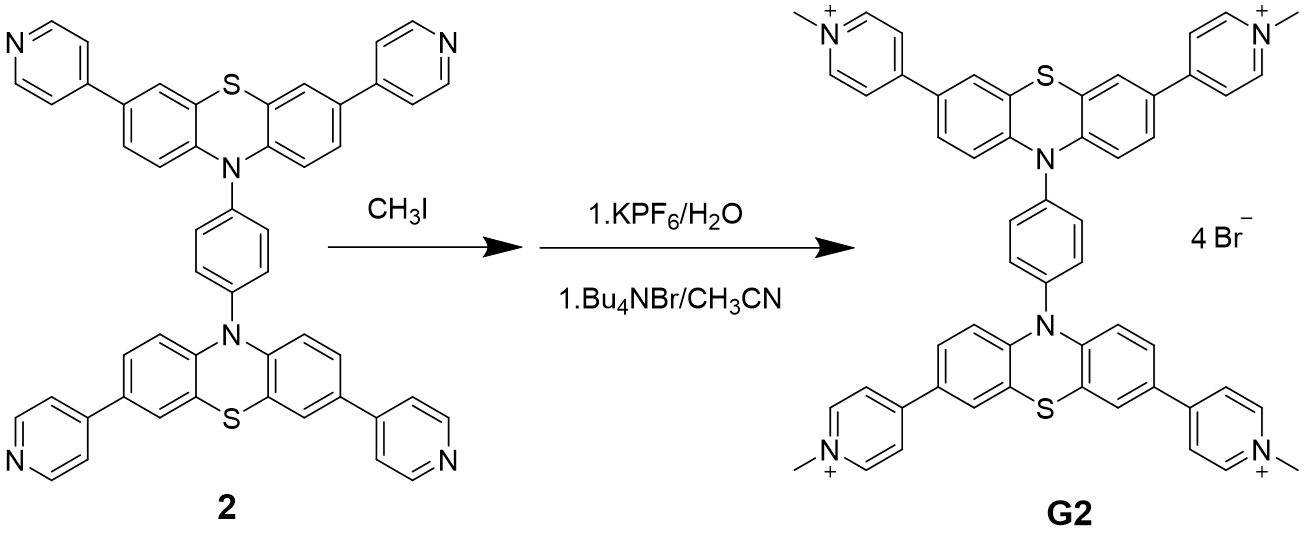


CH_3_I (1 mL) was added to the solution of compound 2^[1]^ (100 mg, 0.13 mmol) in dry DMSO and then stirred at room temperature for 24 h. The above reaction mixture was poured in diethyl ether to obtain the precipitate, which was washed by diethyl ether, and then dissolved in water. The saturated KPF_6_ solution was added to the above solution to give hexafluorophosphate salt of G2 (G2•PF_6_). After that, the CH_3_CN solution of Bu_4_NBr was added to the CH_3_CN solution of G2•PF_6_ to form the dark precipitate, filtrated, and dried to give G2 (58 mg, yield 38.9 %). ^1^H NMR (400 MHz, (CD_3_)_2_SO): δ 8.95 (d, *J* = 6.7 Hz, 8H), 8.43 (d, *J* = 6.6 Hz, 8H), 8.03-7.95 (m, 8H), 7.79 (d, *J* = 8.6 Hz, 4H), 6.45 (d, *J* = 8.7 Hz, 4H), 4.30 (s, 12H). ^13^C NMR (100 MHz, (CD_3_)_2_SO): δ 152.62, 145.88, 145.43, 128.66, 128.54, 126.59, 123.21, 120.37, 117.32, 47.39. HR-MS (ESI): m/z for C_54_H_44_N_6_S_2_Br_4_ calcd. [M-4Br]^4+^= 210.0762, found: 210.0765.


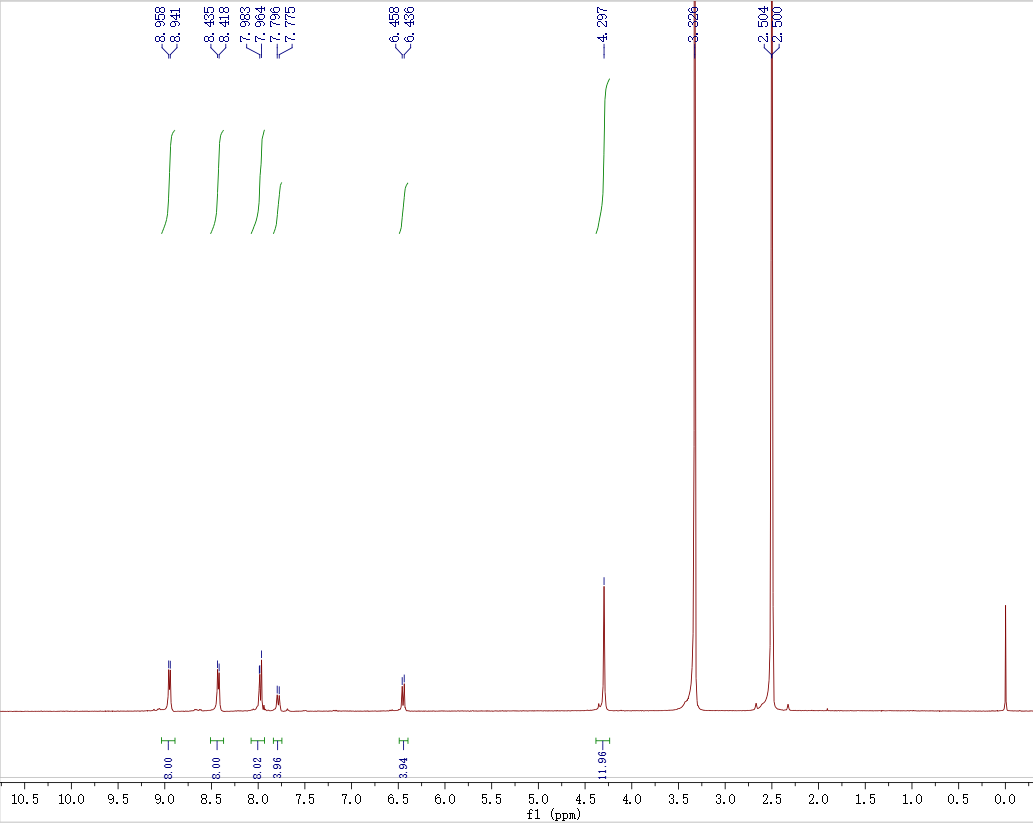


**Figure S4**. ^1^H NMR (400 MHz) spectrum of G2 in (CD_3_)_2_SO at 25 ℃.


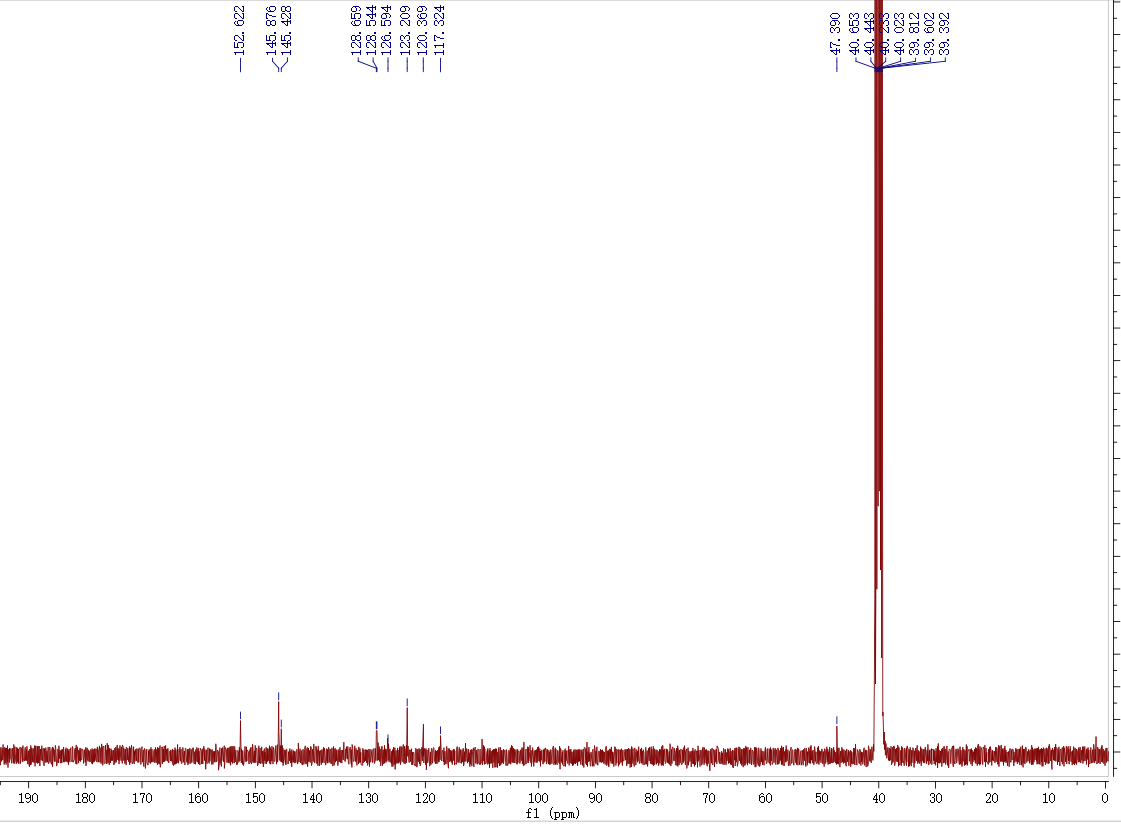


**Figure S5**. ^13^C NMR (100 MHz) spectrum of G2 in (CD_3_)_2_SO at 25 ℃.


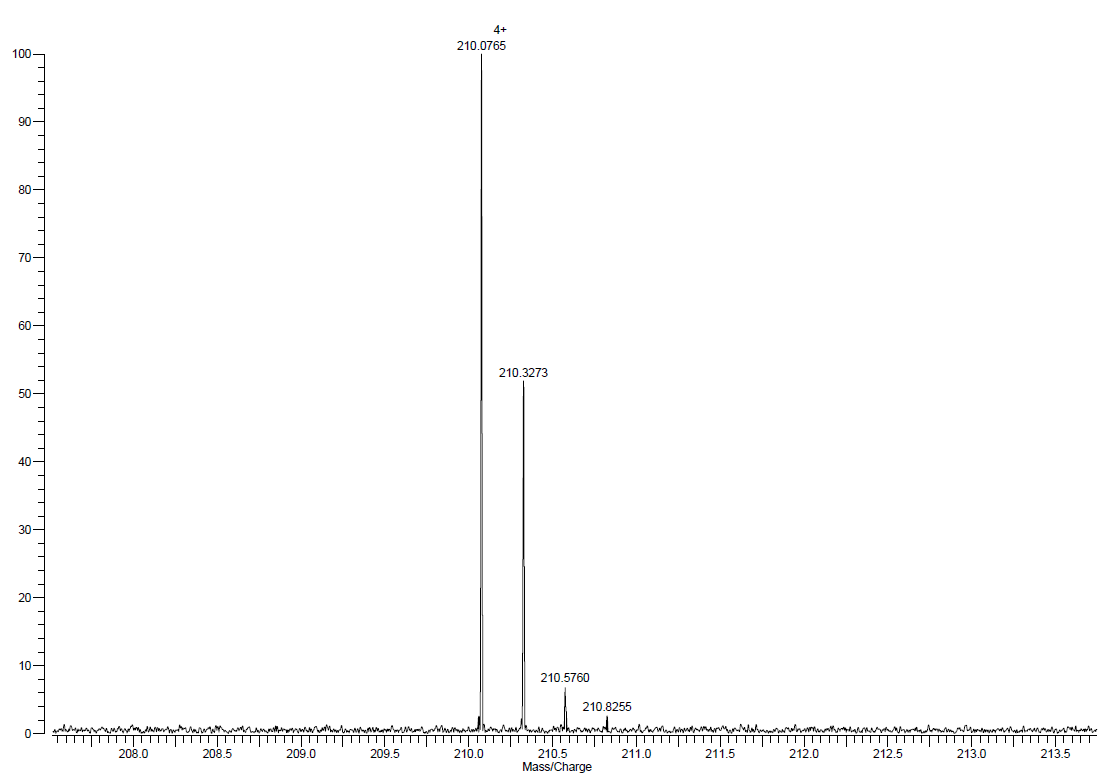


**Figure S6.** HR-MS spectrum of G2.

- 1. **The Synthesis of G3**

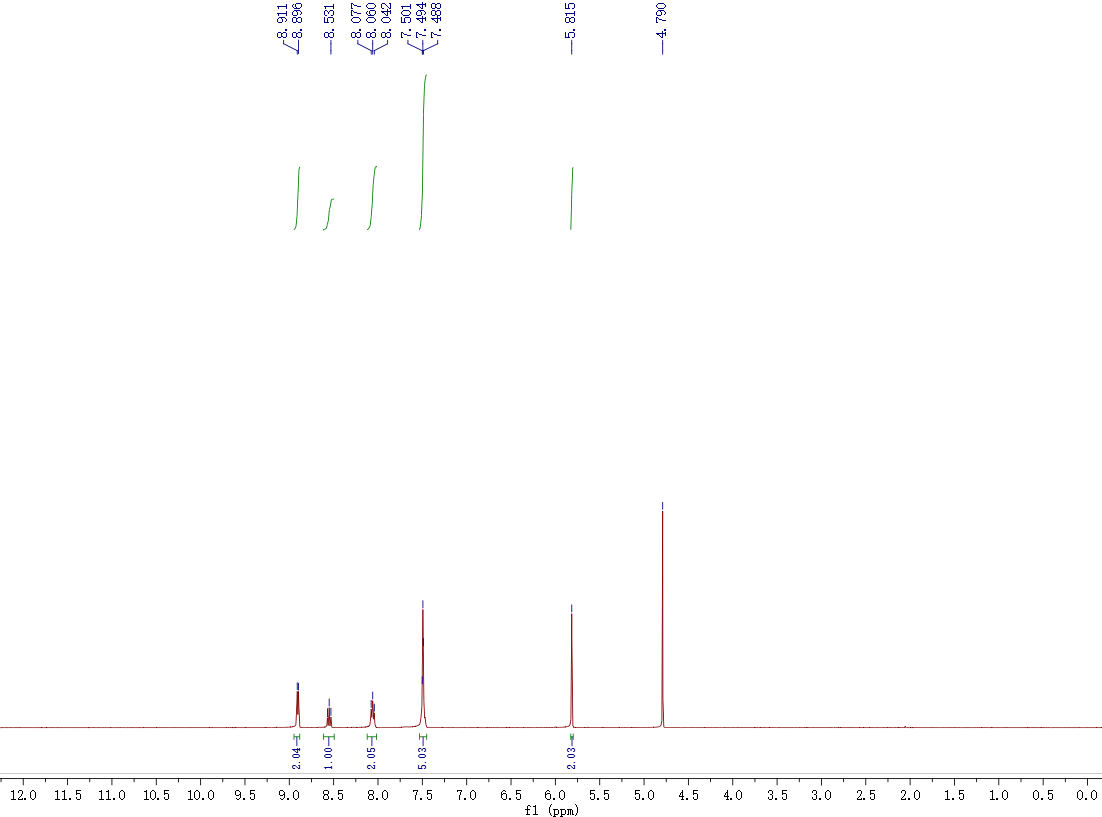


**Figure S7**. ^1^H NMR (400 MHz) spectrum of G3 in D_2_O at 25 ℃.


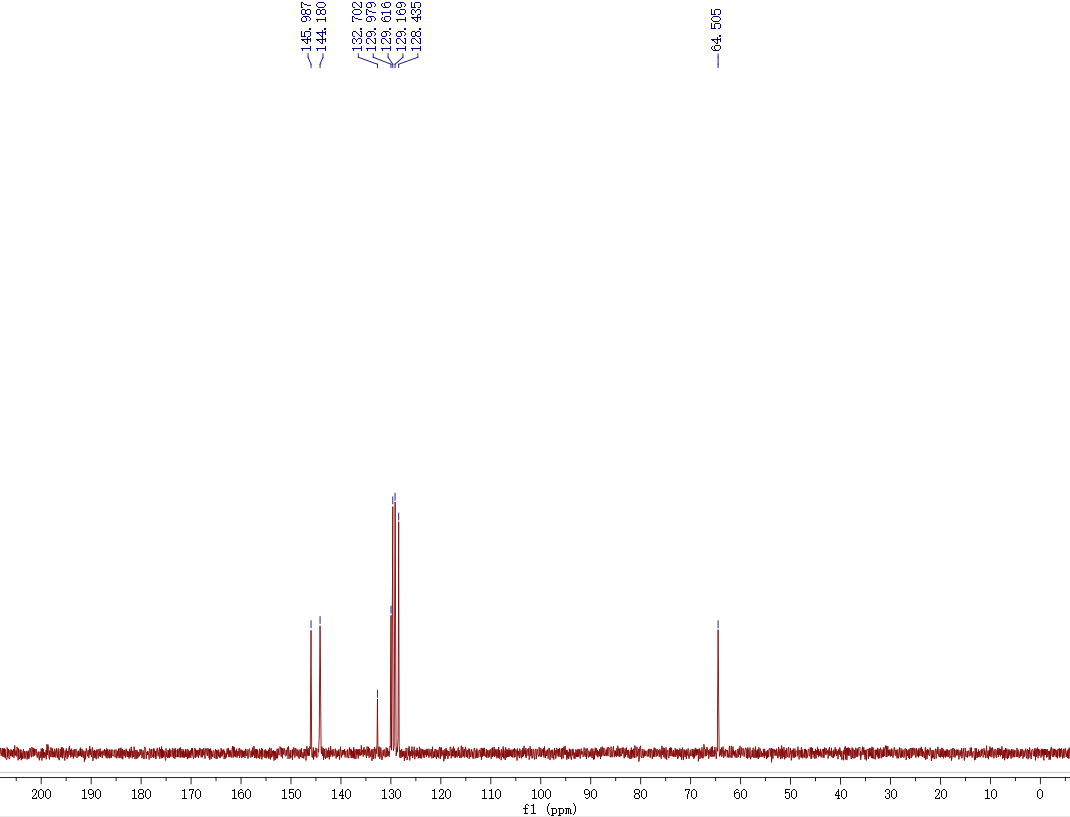


**Figure S8**. ^13^C NMR (100 MHz) spectrum of G3 in D_2_O at 25 ℃.


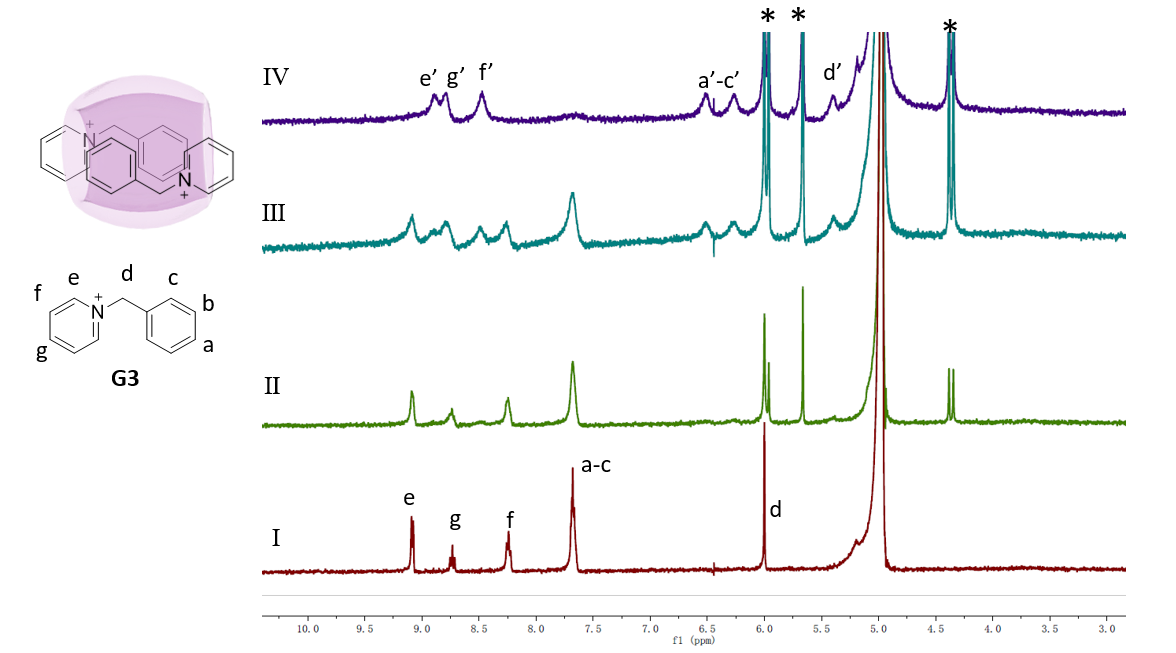


**Figure S9.** ^1^H NMR (400 MHz) spectra of G3 with the addition of CB[8] in D_2_O ([G3] = 1 mM, Ⅰ): 0 eq; Ⅱ):0.1 eq; Ⅲ):0.3 eq; Ⅳ): 0.5 eq CB[8]; *: the protons of CB[8]).


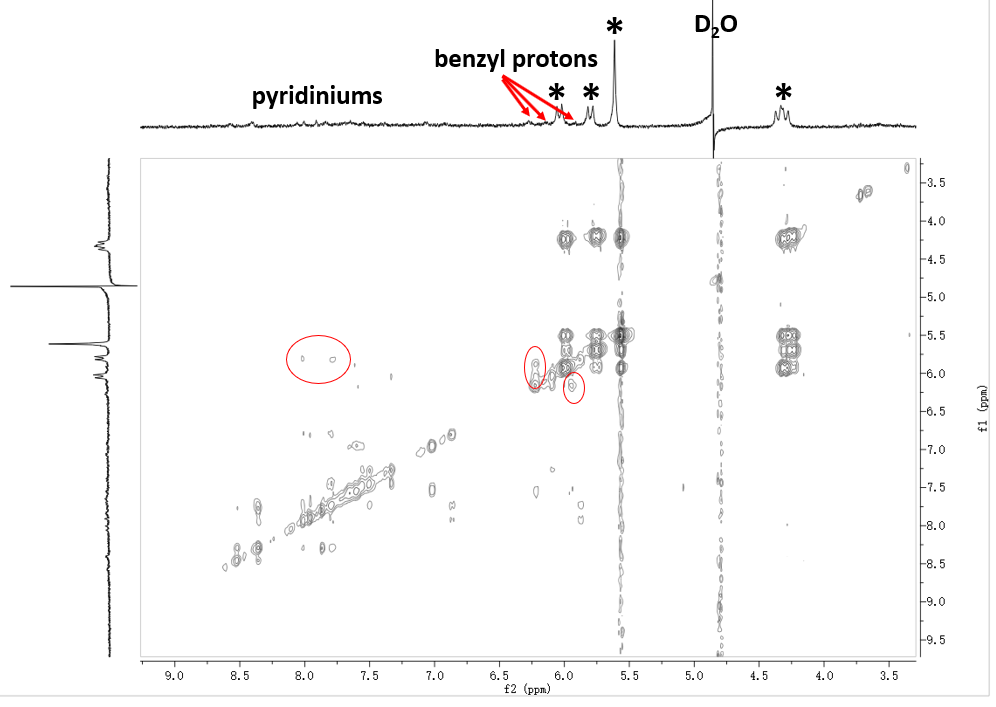


**Figure S10.** 2D NOESY (400 MHz) spectrum of G1/CB[8] in D_2_O/(CD_3_)_2_SO = 95/5 ([G1] = 0.2 mM, [CB[8]] = 0.4 mM; *: the protons of CB[8]) .


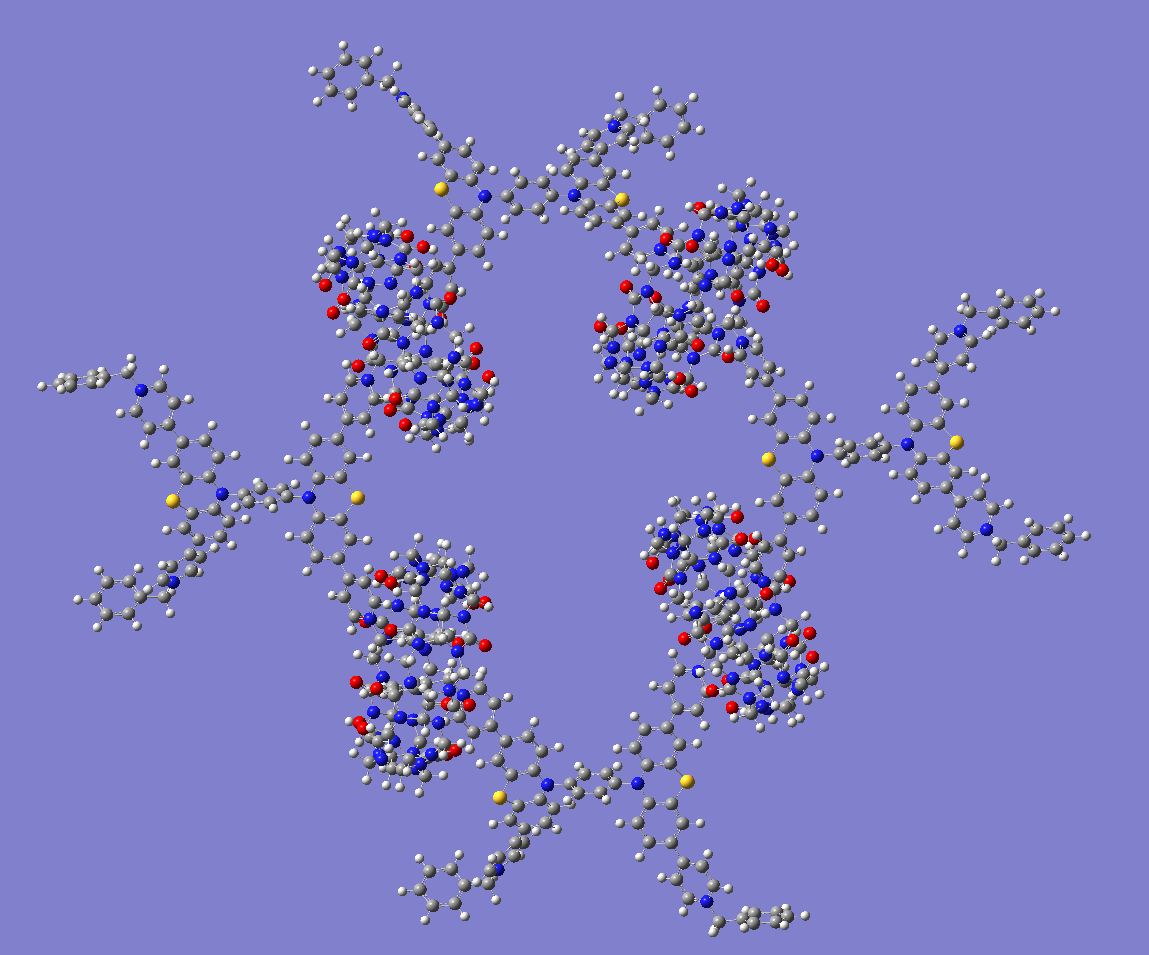


**Figure S11.** Simulated combination mode of G1/CB[8] (Geometry optimization of structure G1/CB[8] was performed using the semi-empirical method AM1 in the Gaussian 16^2^ program).


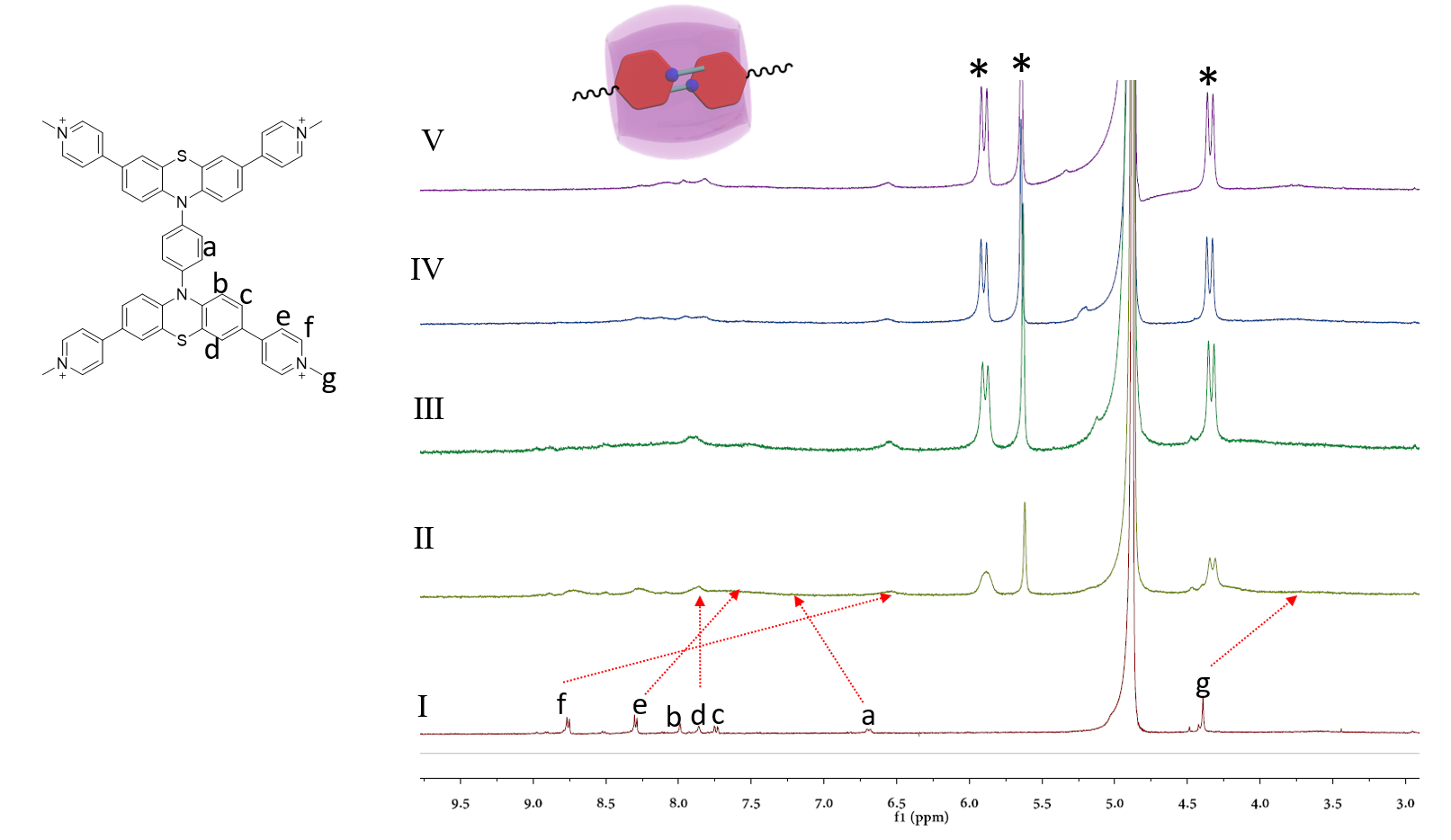


**Figure S12.** ^1^H NMR (400 MHz) spectra of G2 with the addition of CB[8] in D_2_O/(CD_3_)_2_SO = 95/5 ([G2] = 0.2 mM, Ⅰ): 0 eq; Ⅱ):0.5 eq; Ⅲ):1.0 eq; Ⅳ): 1.5 eq; Ⅴ): 2.0 eq CB[8]; *: the protons of CB[8]) .


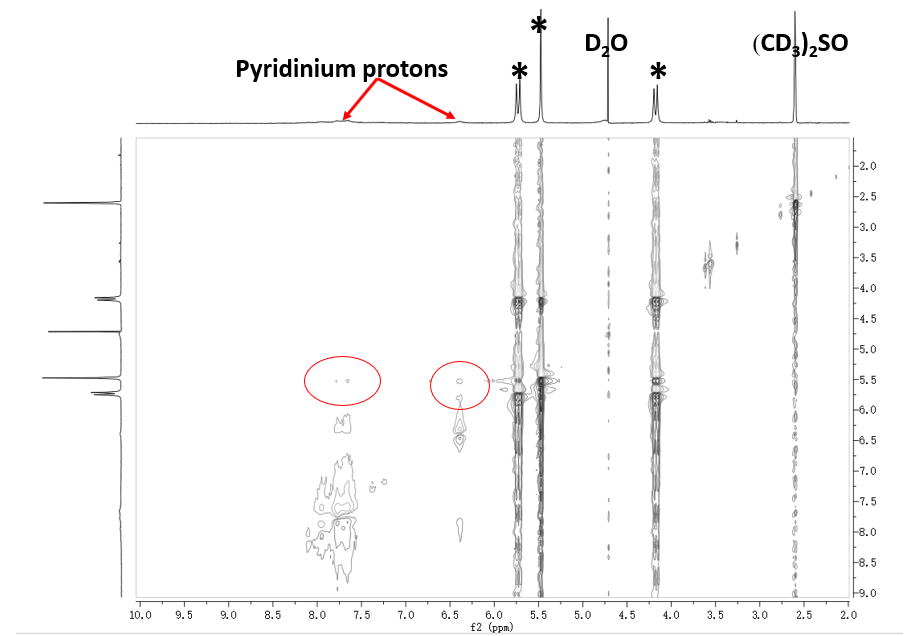


**Figure S13.** 2D NOESY (400 MHz) spectrum of G2/CB[8] in D_2_O/(CD_3_)_2_SO = 95/5 ([G2] = 0.2 mM, [CB[8]] = 0.4 mM; *: the protons of CB[8]).


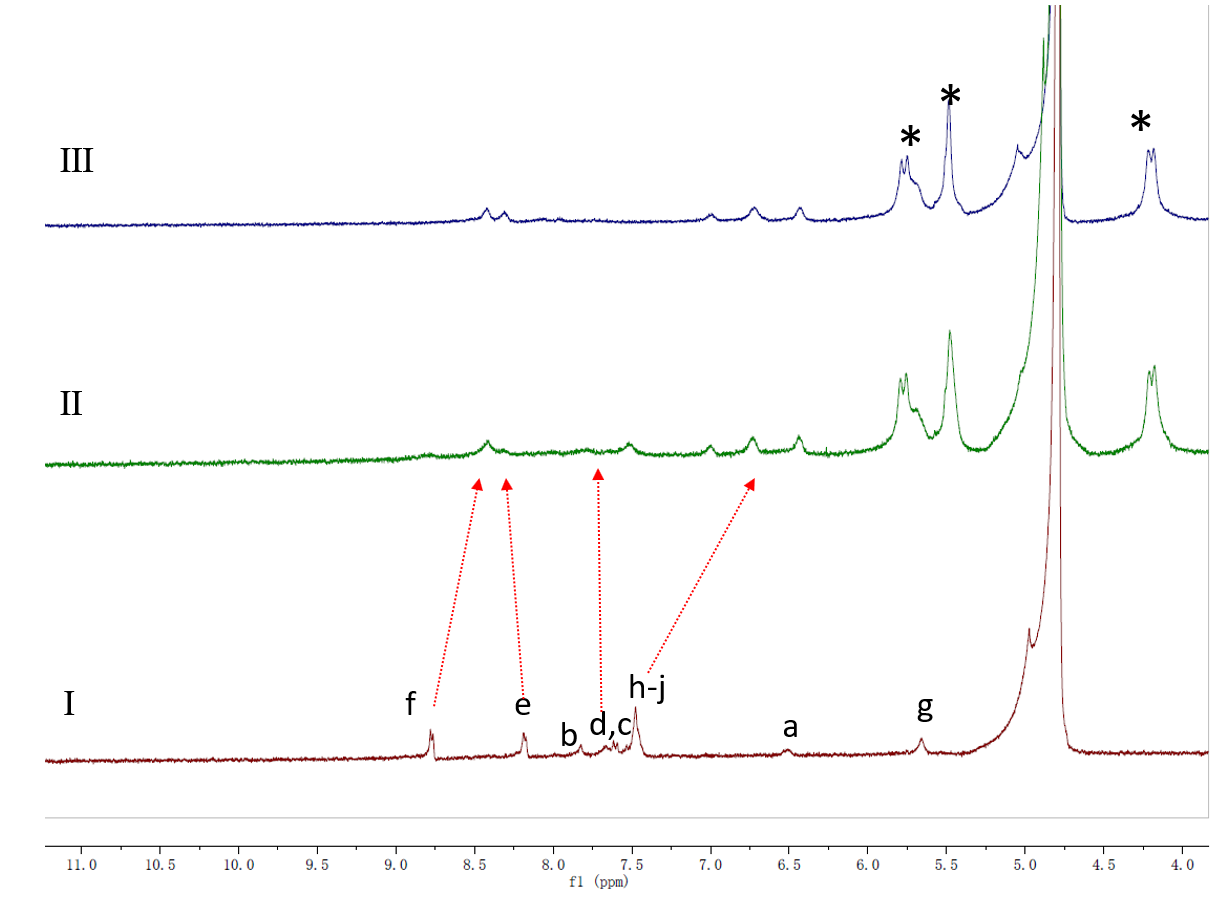


**Figure S14.** ^1^H NMR (400 MHz) spectra of G1 with the addition of CB[7] in D_2_O/(CD_3_)_2_SO = 95/5 ([G1] = 0.2 mM, Ⅰ): 0 eq; Ⅱ): 2.0 eq; Ⅲ): 4.0 eq CB[7]; *: the protons of CB[7]).


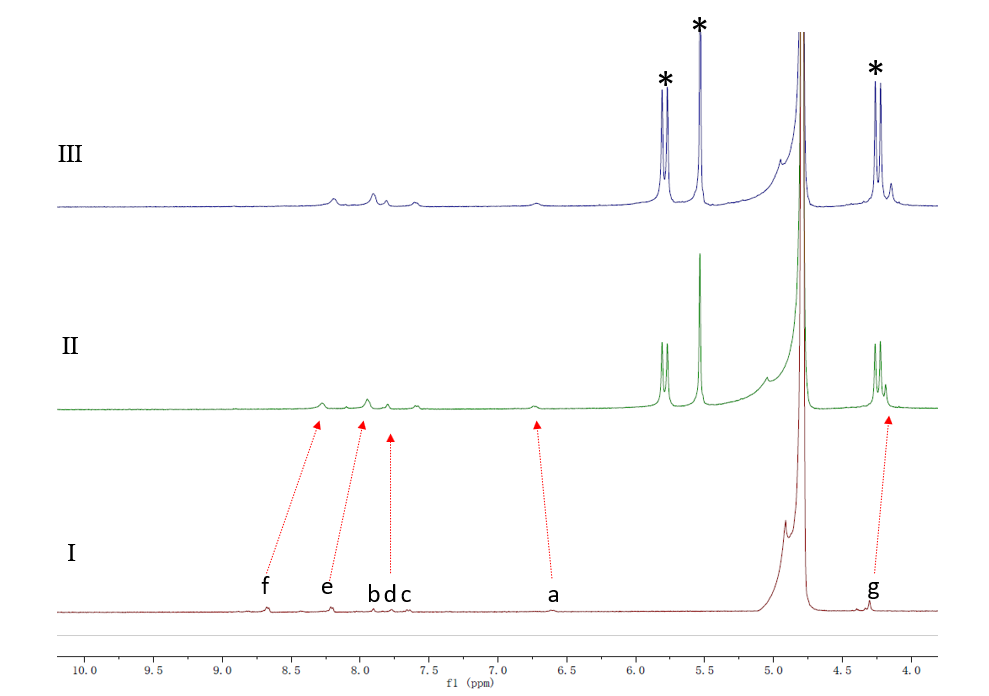


**Figure S15.** ^1^H NMR (400 MHz) spectra of G2 with the addition of CB[7] in D_2_O/(CD_3_)_2_SO = 95/5 ([G2] = 0.2 mM, Ⅰ): 0 eq; Ⅱ): 2.0 eq; Ⅲ): 4.0 eq CB[7]; *: the protons of CB[7]).


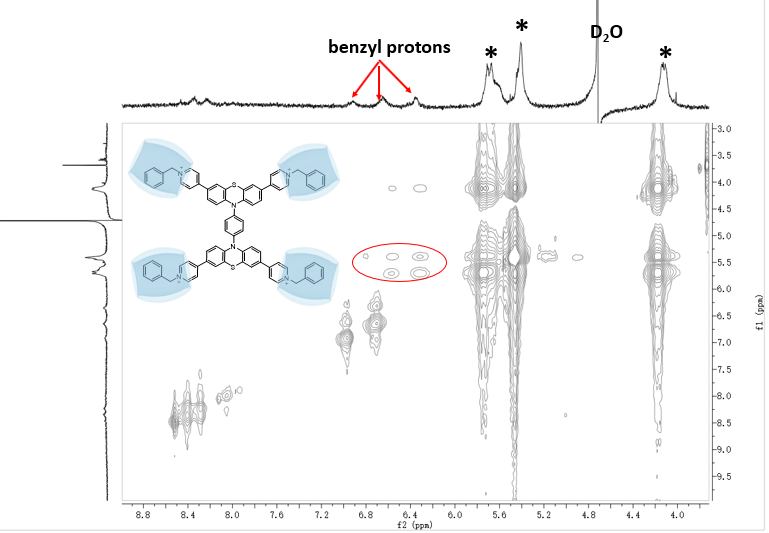


**Figure S16.** 2D NOESY (400 MHz) spectrum of G1/CB[7] in D_2_O/(CD_3_)_2_SO = 95/5 ([G1] = 0.2 mM, [CB[7]] = 0.8 mM; *: the protons of CB[7]).


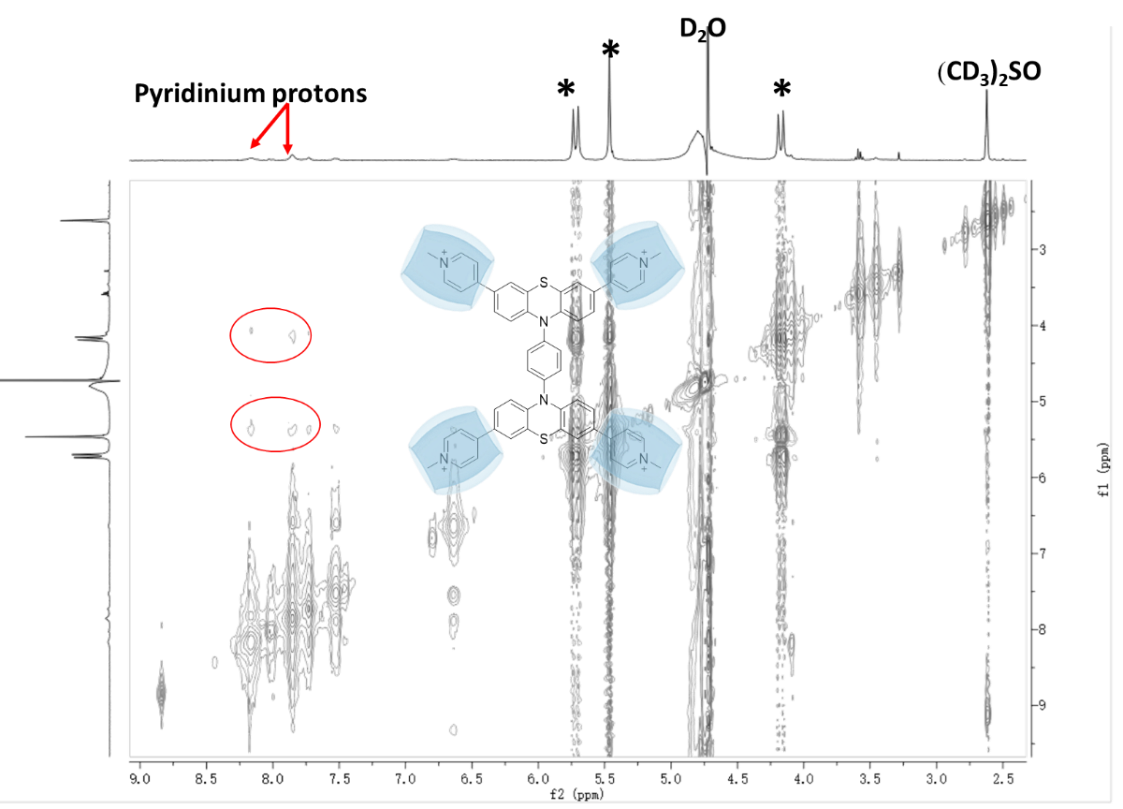


**Figure S17.** 2D NOESY (400 MHz) spectrum of G2/CB[7] in D_2_O/(CD_3_)_2_SO = 95/5 ([G2] = 0.2 mM, [CB[7]] = 0.8 mM; *: the protons of CB[7]).


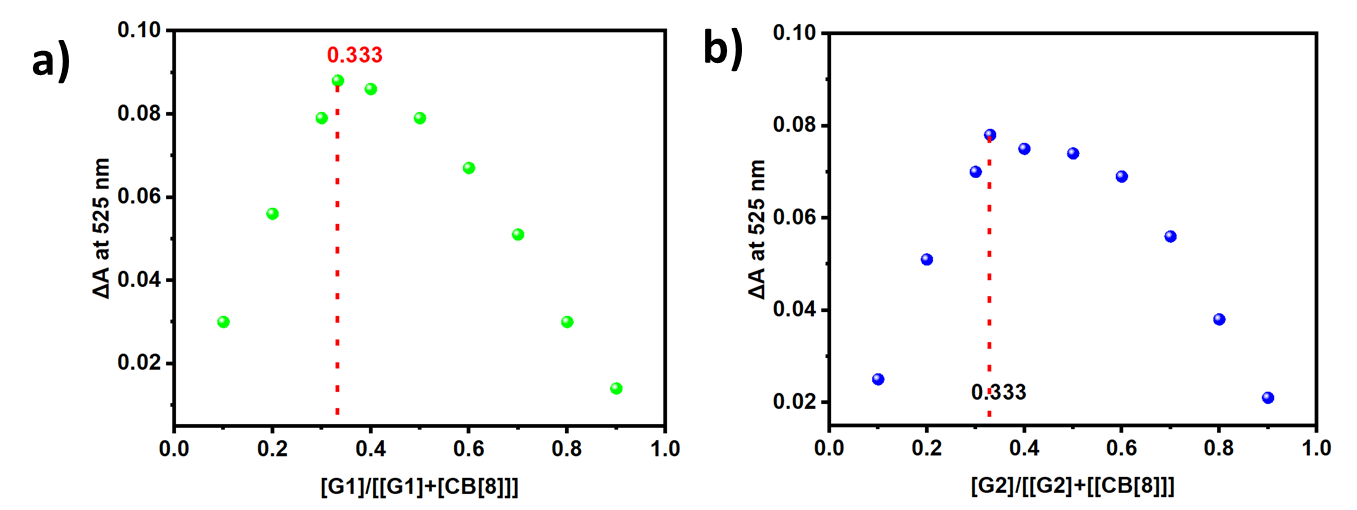


**Figure S18.** Job plot of a) G1/CB[8] and b) G2/CB[8] ([G1] = [G2] = [CB[8]] = 0.02 mM).

**Calculation of the binding constant**

The binding constant (*K*) for a stoichiometric 1:2 complex G/CB[8] (G: CB[8] = 1:2) of G with CB[8] was calculated by using the non-linear least-squares fit of the titration data according to the following formula with the Origin program^3-4^


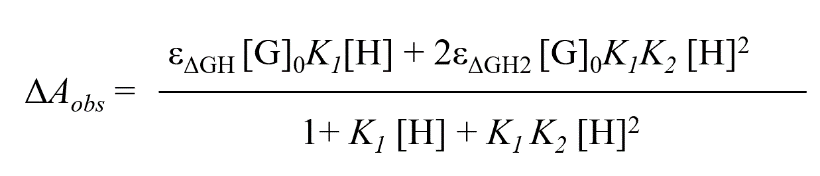


Δ*A_obs_* is the UV-vis absorption change of G upon the addition of CB[8]. *K_1_* and *K_2_* are the first-order and the second-order binding constants, respectively. ε_ΔGH_ is the molar absorption coefficient change between the G/CB[8] complex and G. ε_ΔGH2_ is the molar absorption coefficient change between the G/CB[8]_2_ complex and G. [G]_0_ is the initial concentration of guest. [H] is the concentration of the free host.


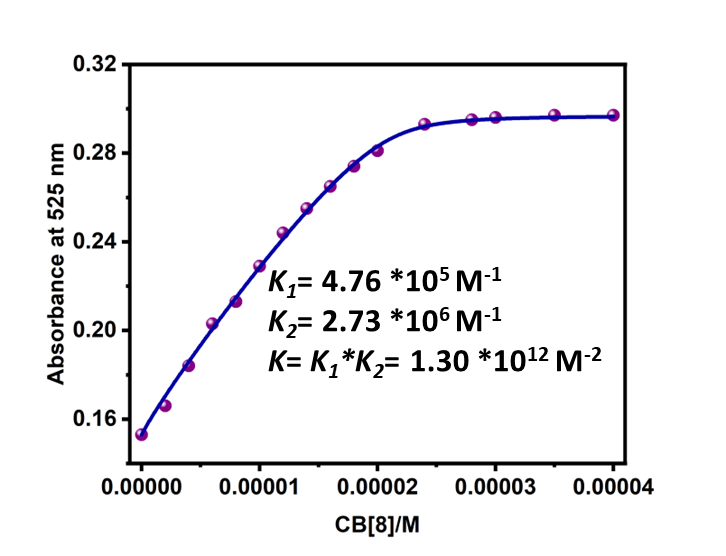


**Figure S19.** Nonlinear least-squares analysis of the absorbance intensity changes of G1 with the addition of CB[8] to calculate the *Ks* value between G1 and CB[8].


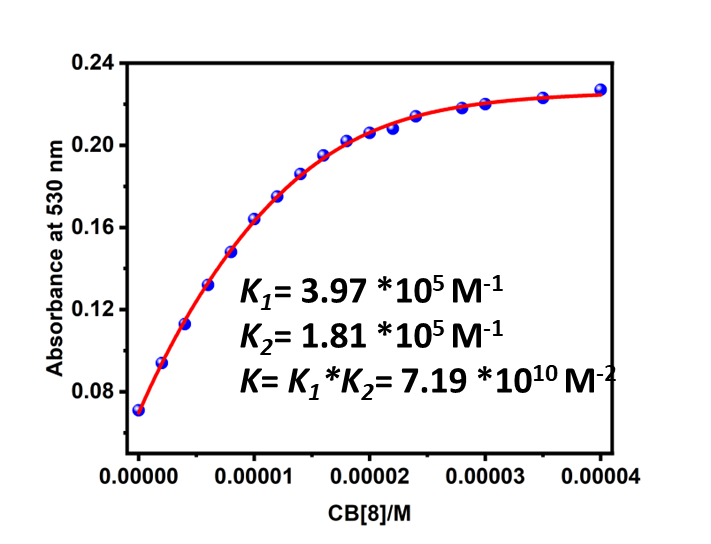


**Figure S20.** Nonlinear least-squares analysis of the absorbance intensity changes of G2 with the addition of CB[8] to calculate the *Ks* value between G2 and CB[8].


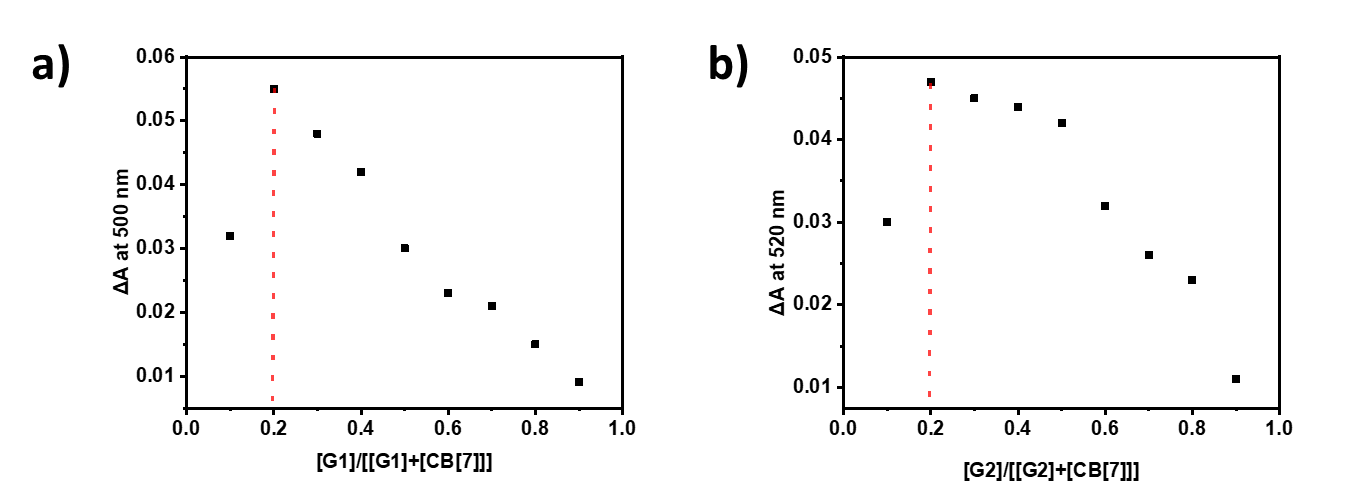


**Figure S21.** Job plot of a) G1/CB[7] and b) G2/CB[7] ([G1] = [G2] = [CB[7]] = 0.04 mM).


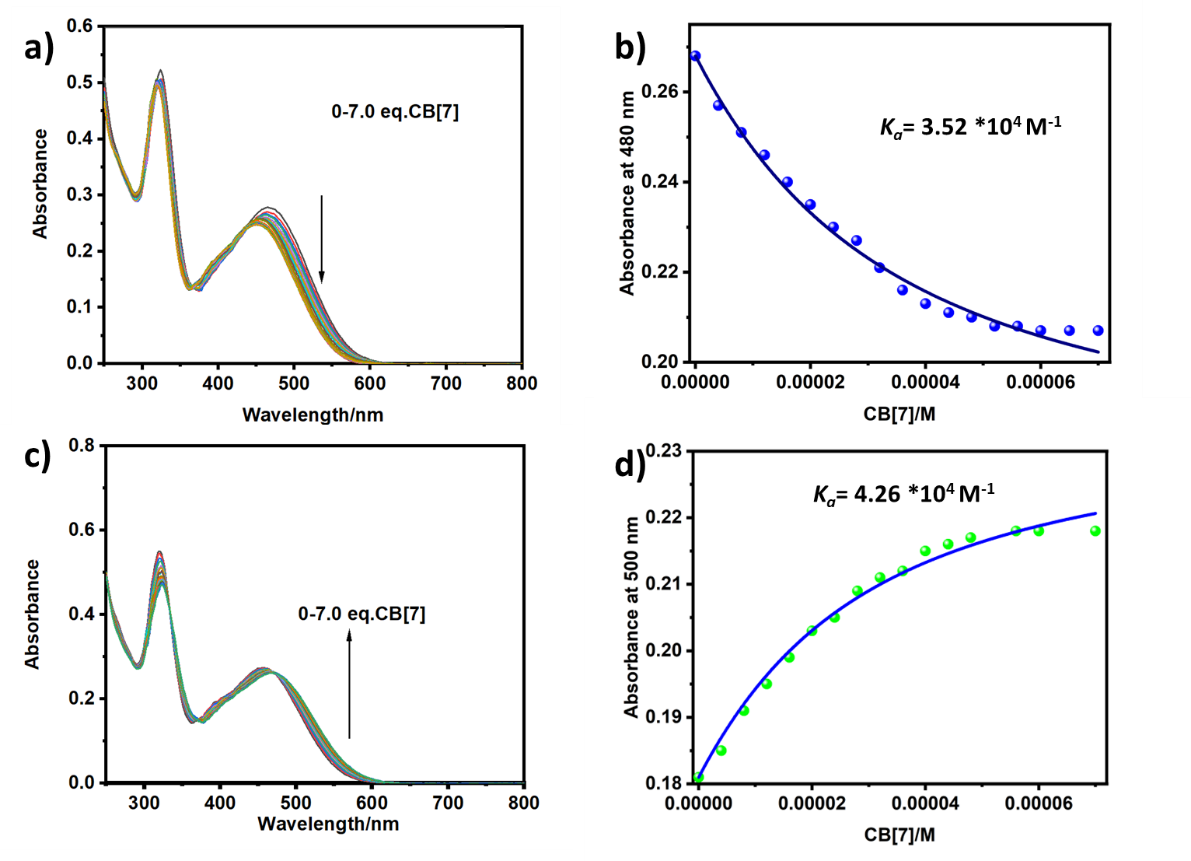


**Figure S22.** a) UV-vis spectra of G1 in the presence of CB[7]. b) Nonlinear least-squares analysis of the absorbance intensity changes of G1 with the addition of CB[7] to calculate the apparent binding constant (*K_a_*) value between G1 and CB[7]. c) UV-vis spectra of G2 in the presence of CB[7]. d) Nonlinear least-squares analysis of the absorbance intensity changes of G2 with the addition of CB[7] to calculate the apparent binding constant (*K_a_*) value between G2 and CB[7] ([G1] = [G2] = 0.01 mM).


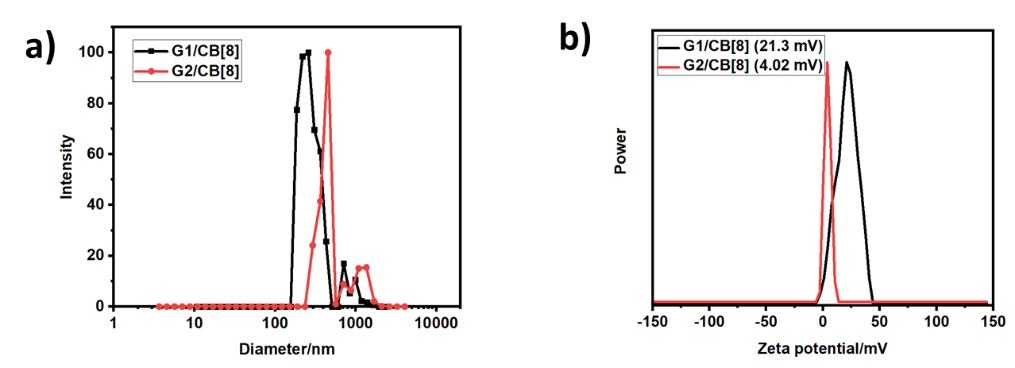


**Figure S23.** a) DLS and b) zeta potential of G1/CB[8] and G2/CB[8].


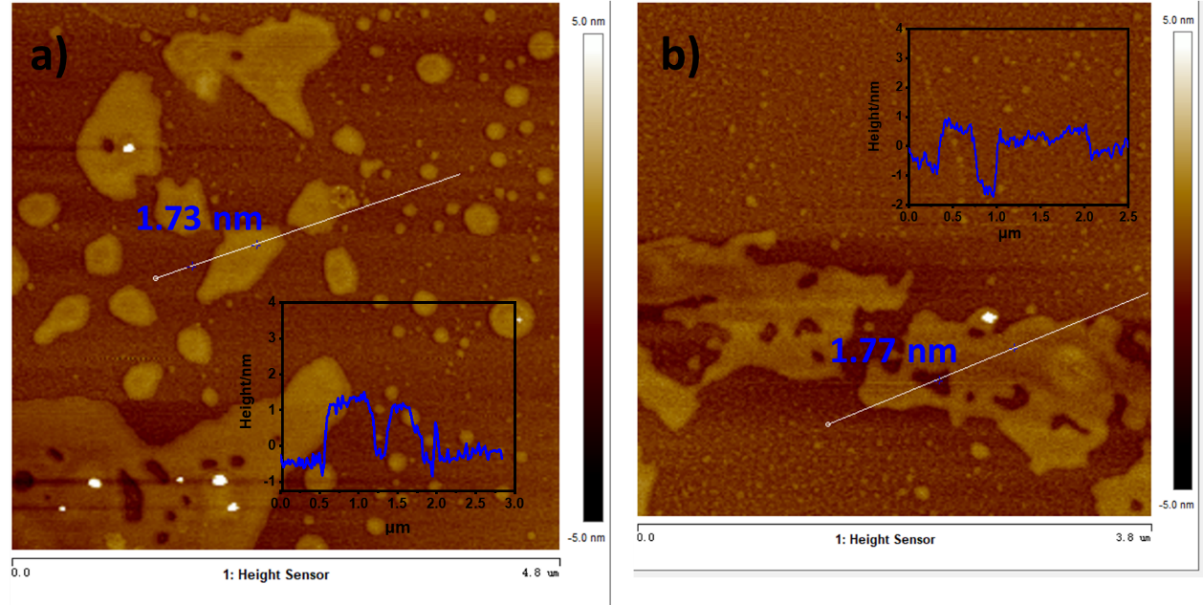


**Figure S24.** AFM images of a) G1/CB[8] and b) G2/CB[8] (spin-coating on the mica lamination).


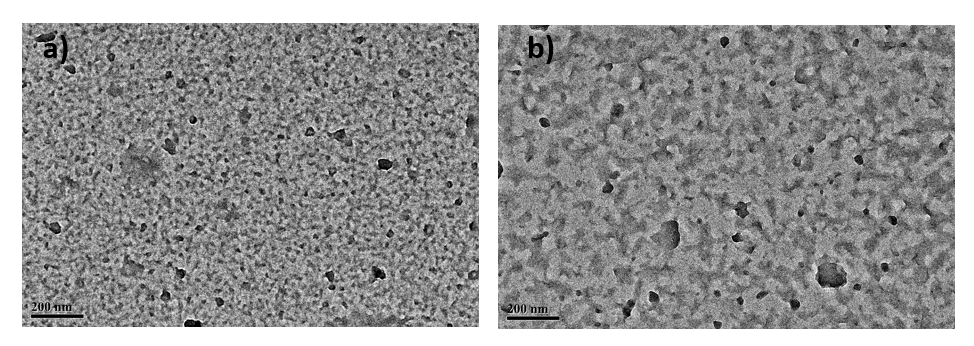


**Figure S25.** TEM images of a) G1/CB[7] and b) G2/CB[7].


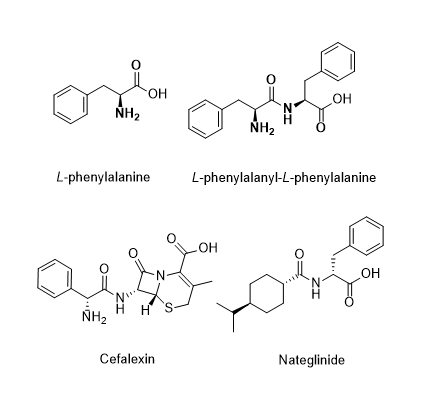


**Figure S26.** Structure of *L*-phenylalanine (*L*-Phe), *L*-phenylalanyl-*L*-phenylalanine (*L*-FF), Cefalexin, and Nateglinide.


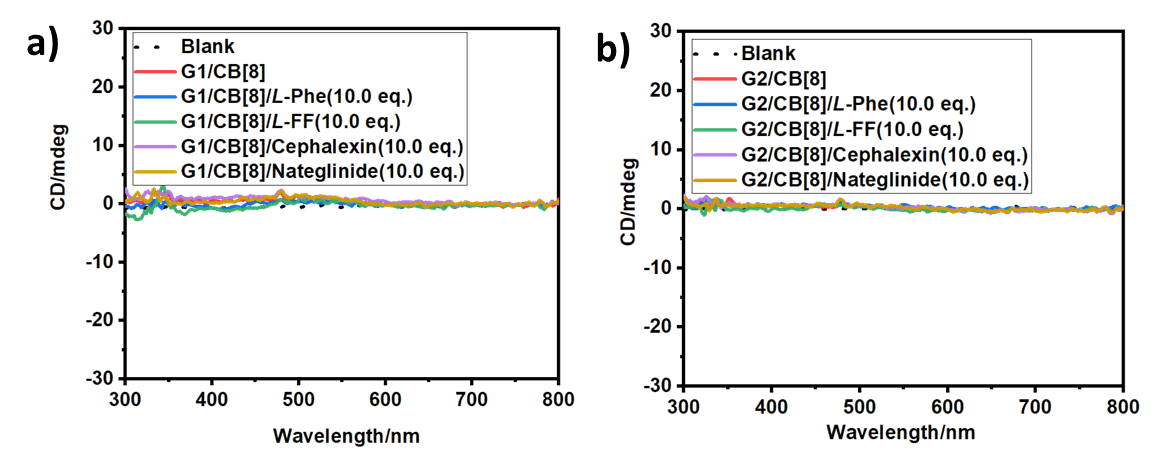


**Figure S27.** (a) G1/CB[8] and (b) G2/CB[8] with the addition of *L*-Phe, *L*-FF, Cephalexin, and Nateglinide.


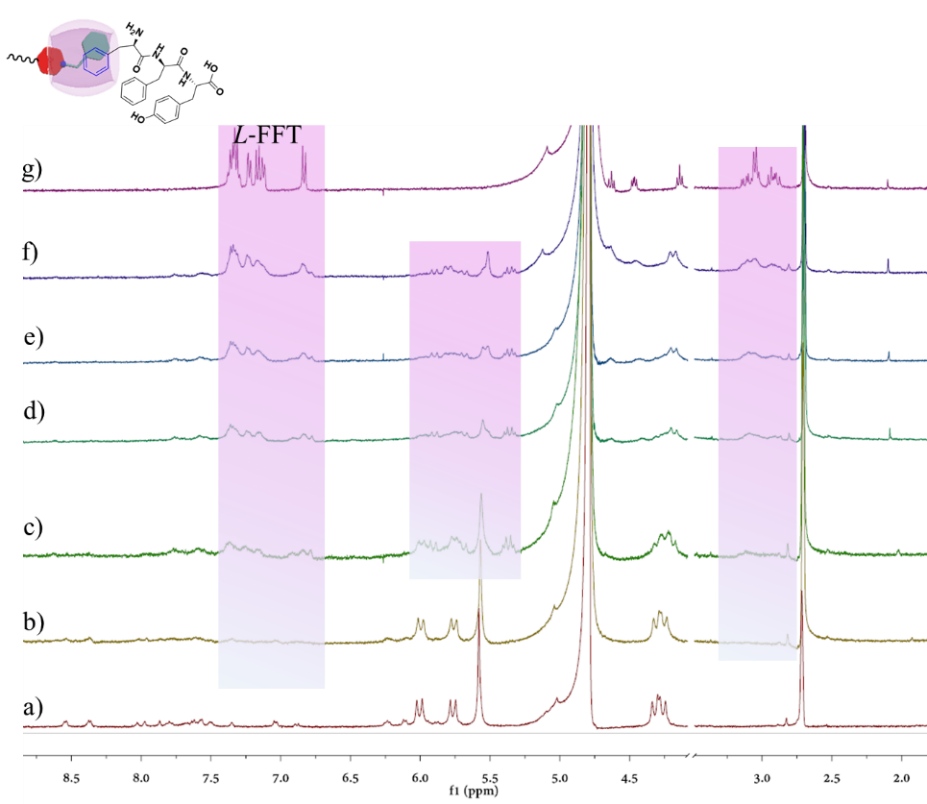


**Figure S28.** ^1^H NMR (400 MHz) spectra of G1/CB[8] with the addition of *L*-FFT in D_2_O/(CD_3_)_2_SO = 95/5. ([G1] = 0.2 mM, [CB[8]]= 0.4 mM, a): 0 eq; b): 2.0 eq; c): 4.0 eq; d): 6.0 eq; e): 8.0 eq; f):10.0 eq; *L*-FFT; g) 2.0 mM *L*-FFT alone).


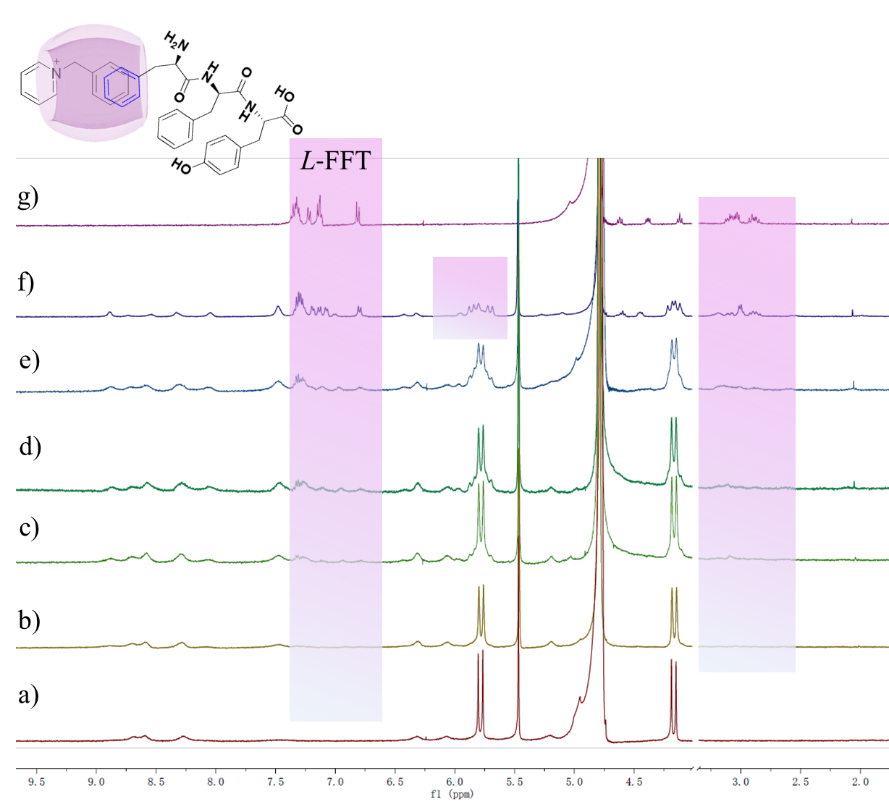


**Figure S29.** ^1^H NMR (400 MHz) spectra of G3/CB[8] with the addition of *L*-FFT in D_2_O. ([G3] = 1 mM, [CB[8]] = 0.5 mM, a): 0 eq; b): 0.2 eq; c): 0.4 eq; d): 0.6 eq; e): 0.8 eq; f): 1.0 eq; *L*-FFT; g) 1.0 mM *L*-FFT alone).


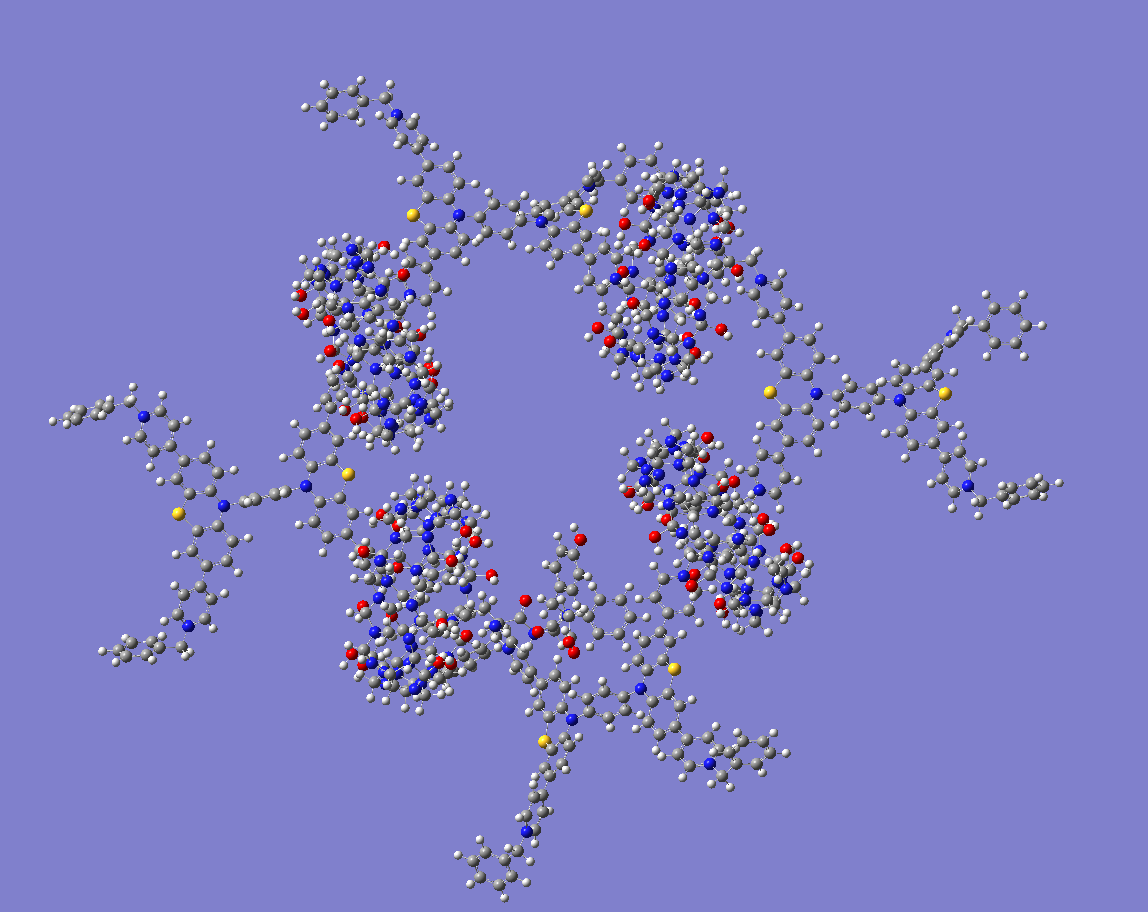


**Figure S30.** Simulated combination mode of G1/CB[8]/*L*-FFT (Only one CB[8] encapsulated tripeptide was shown in the combination mode for clarity. Geometry optimization of structure G1/CB[8]/*L*-FFT was performed using the semi-empirical method AM1 in the Gaussian 16^2^ program).


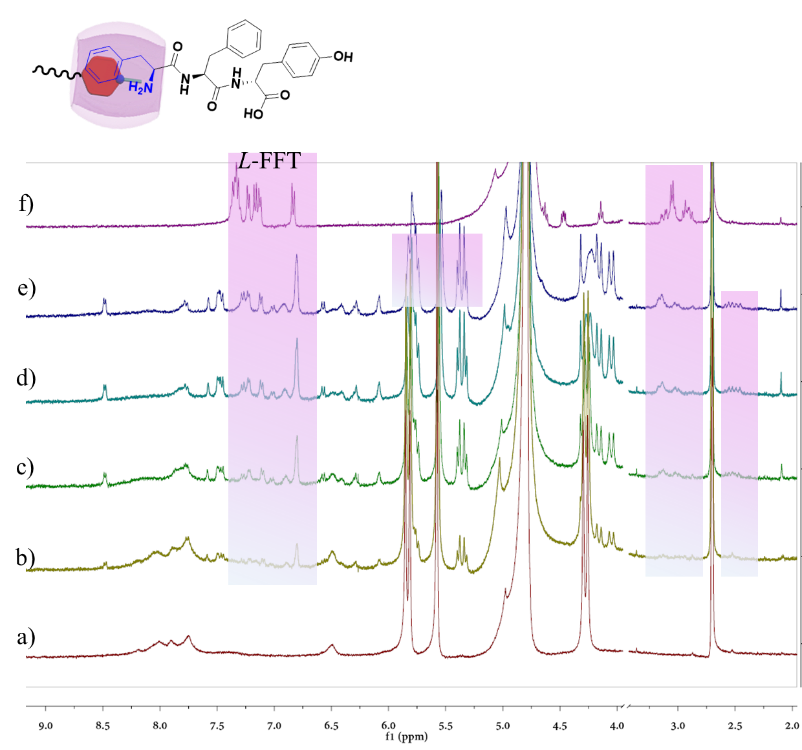


**Figure S31.** ^1^H NMR (400 MHz) spectra of G2/CB[8] with the addition of *L*-FFT in D_2_O ([G] = 0.2 mM, [CB[8]] = 0.4 mM, a): 0 eq; b): 1.0 eq; c): 2.0 eq; d): 3.0 eq; e): 4.0 eq. *L*-FFT; f): 0.8 mM *L*-FFT alone).


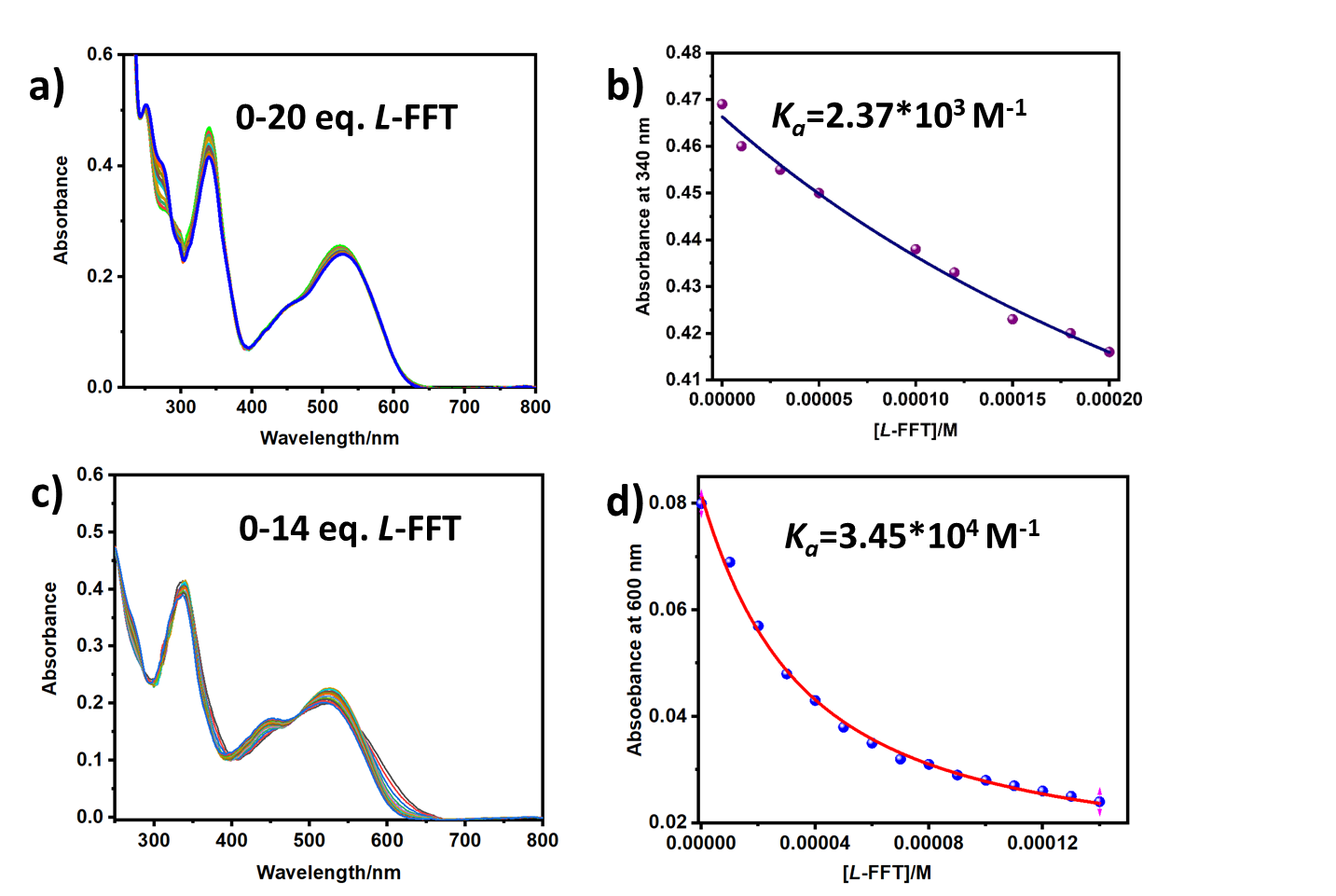


**Figure S32.** a) UV-vis spectra of G1/CB[8] in the presence of *L*-FFT ([[G1]] = 0.01 mM, [CB[8]] = 0.02 mM). b) Nonlinear least-squares analysis of the absorbance intensity changes of G1/CB[8] with the addition of *L*-FFT to calculate the apparent binding constant (*K_a_*) value between G1/CB[8] and *L*-FFT. c) UV-vis spectra of G2/CB[8] in the presence of *L*-FFT ([G2] = 0.01 mM, [CB[8]] = 0.02 mM). d) Nonlinear least-squares analysis of the absorbance intensity changes of G2/CB[8] with the addition of *L*-FFT to calculate the apparent binding constant (*K_a_*) value between G2/CB[8] and *L*-FFT.


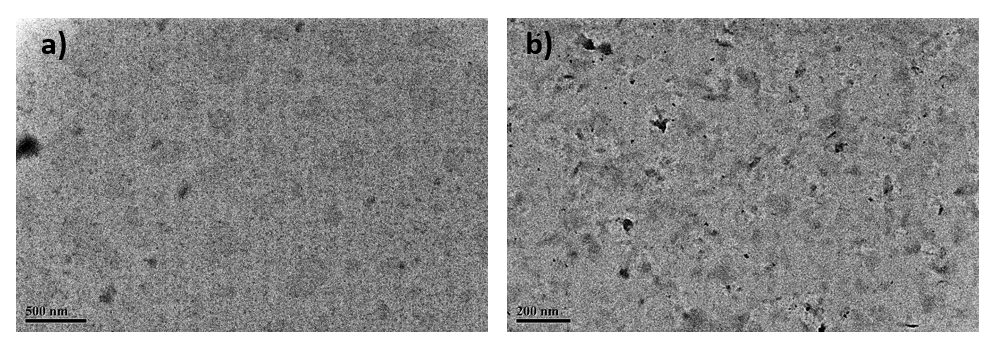


**Figure S33.** TEM images of a) G1/CB[8]/*L-*FFT and b) G2/CB[8]/*L-*FFT.


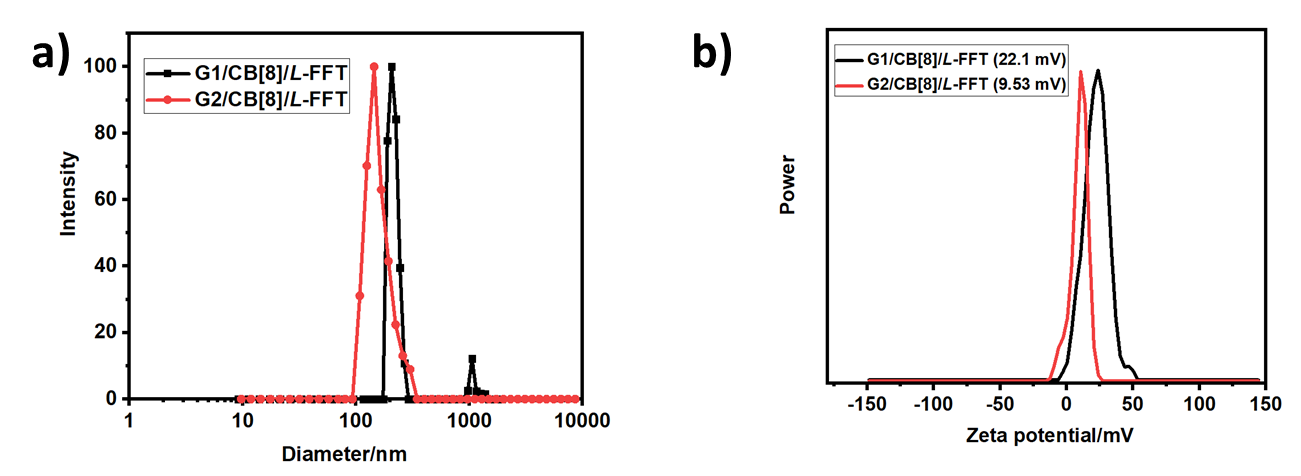


**Figure S34.** a) DLS and b) Zeta potential of G1/CB[8]/*L-*FFT and G2/CB[8]/*L-*FFT.


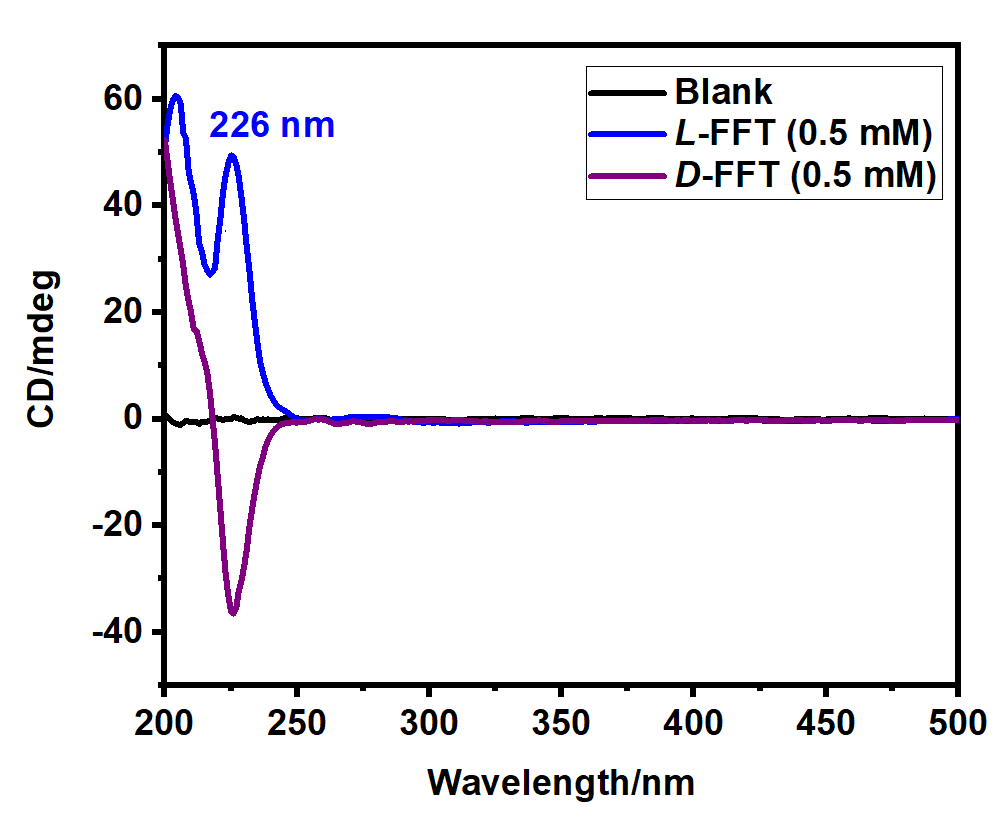


**Figure S35.** The CD spectra of *L*-FFT and *D*-FFT.


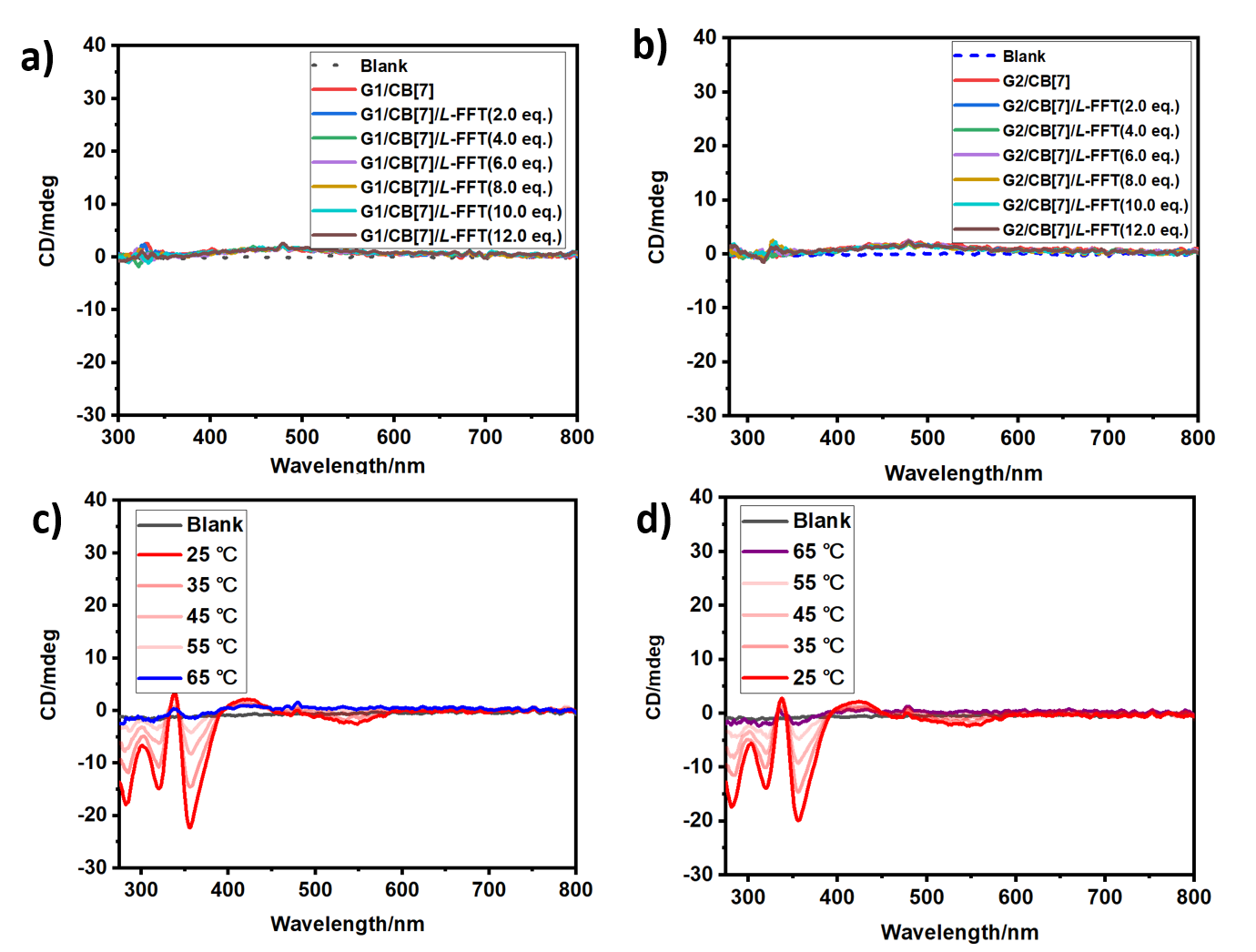


**Figure S36.** The CD spectra of a) G1/CB[7]/*L*-FFT and b) G2/CB[7]/*L*-FFT (([G1] = [G2] = 0.05 mM, [CB[7]] = 0.2 mM). The CD spectra of G2/CB[8]/*L*-FFT c) heated and d) cooled at different temperatures([G2] = 0.05 mM, [CB[8]] = 0.1 mM, [*L*-FFT] = 0.2 mM).


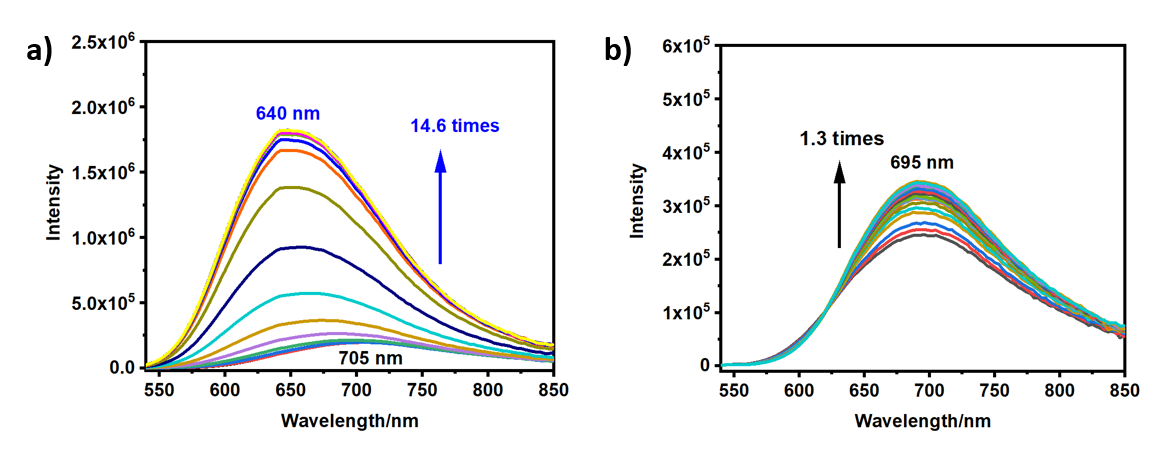


**Figure S37.** a) Fluorescence spectra of G1 in the presence of 0-6.0 eq. CB[7] ([G1] = 0.01 mM, λ_ex_ = 468 nm). b) Fluorescence spectra of G2 in the presence of 0-6.0 eq. CB[7] ([G2] = 0.01 mM, λ_ex_ = 455 nm).


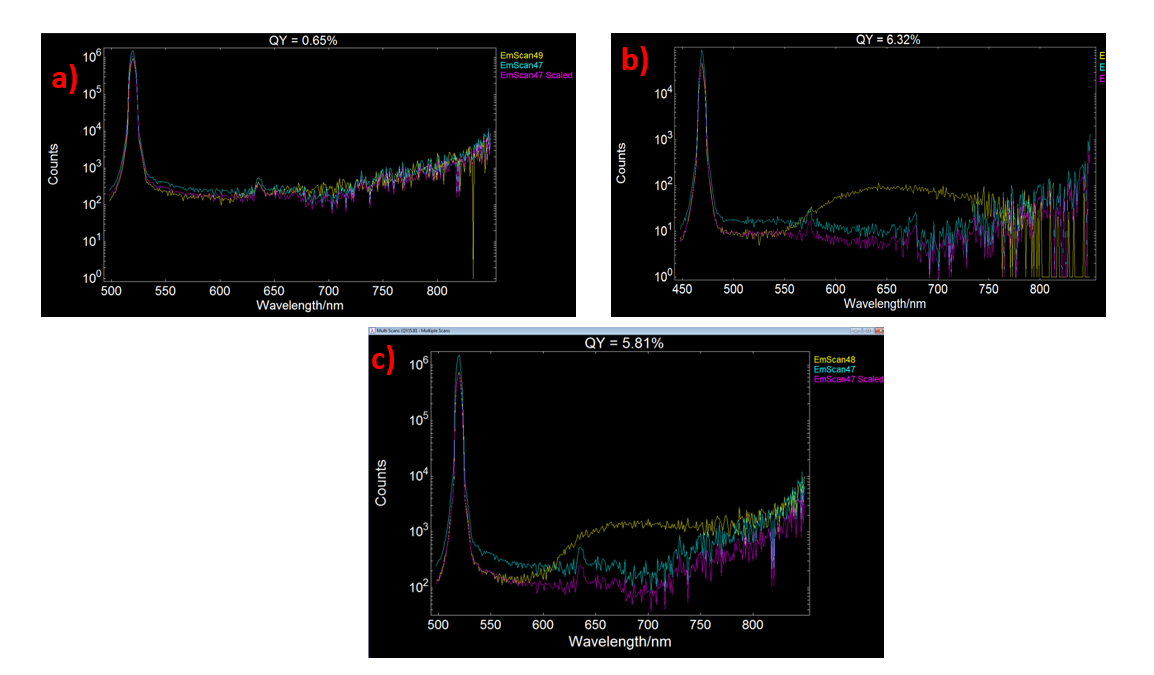


**Figure S38.** The QY of a) G1 (0.65%), b) G1/CB[7] (6.32%), and c) G1/CB[8] (5.81%).


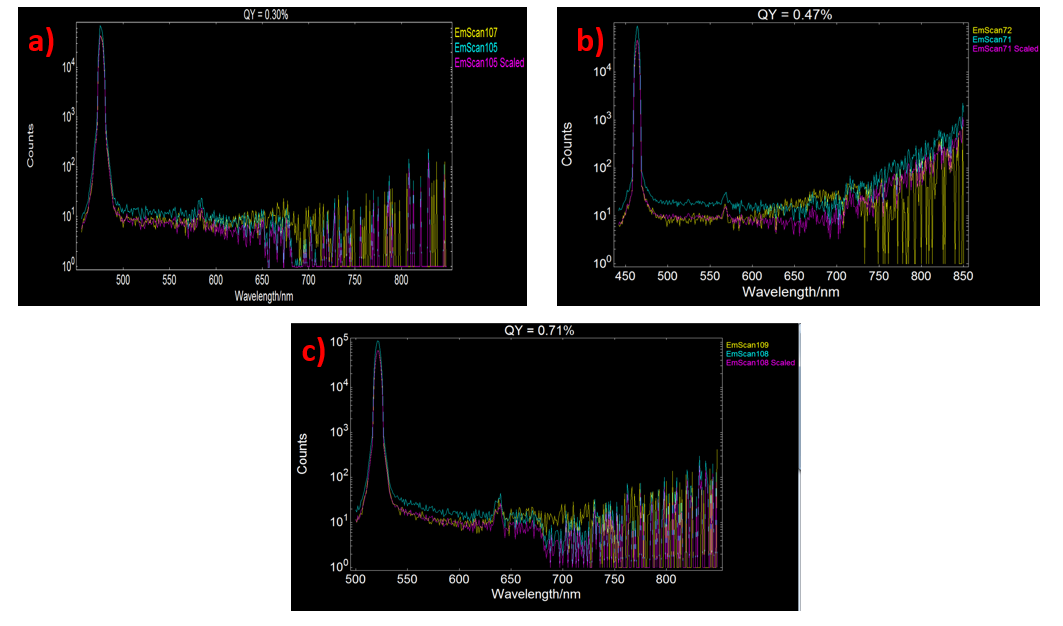


**Figure S39.** The QY of a) G2 (0.30%), b) G2/CB[7] (0.47%), and c) G2/CB[8](0.71%).


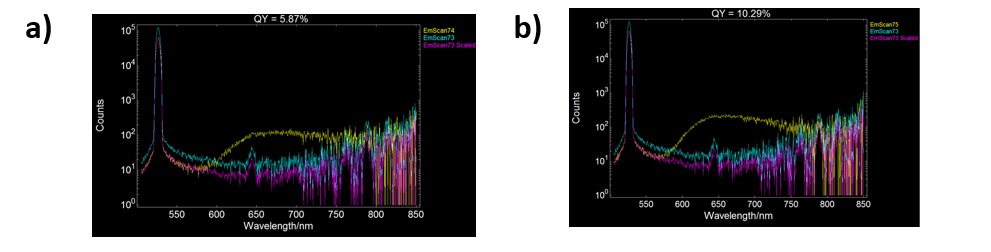


**Figure S40.** QY of a) G1/CB[8]/*L-*FFT (5.87%) and b) G2/CB[8]/*L-*FFT (10.29%).


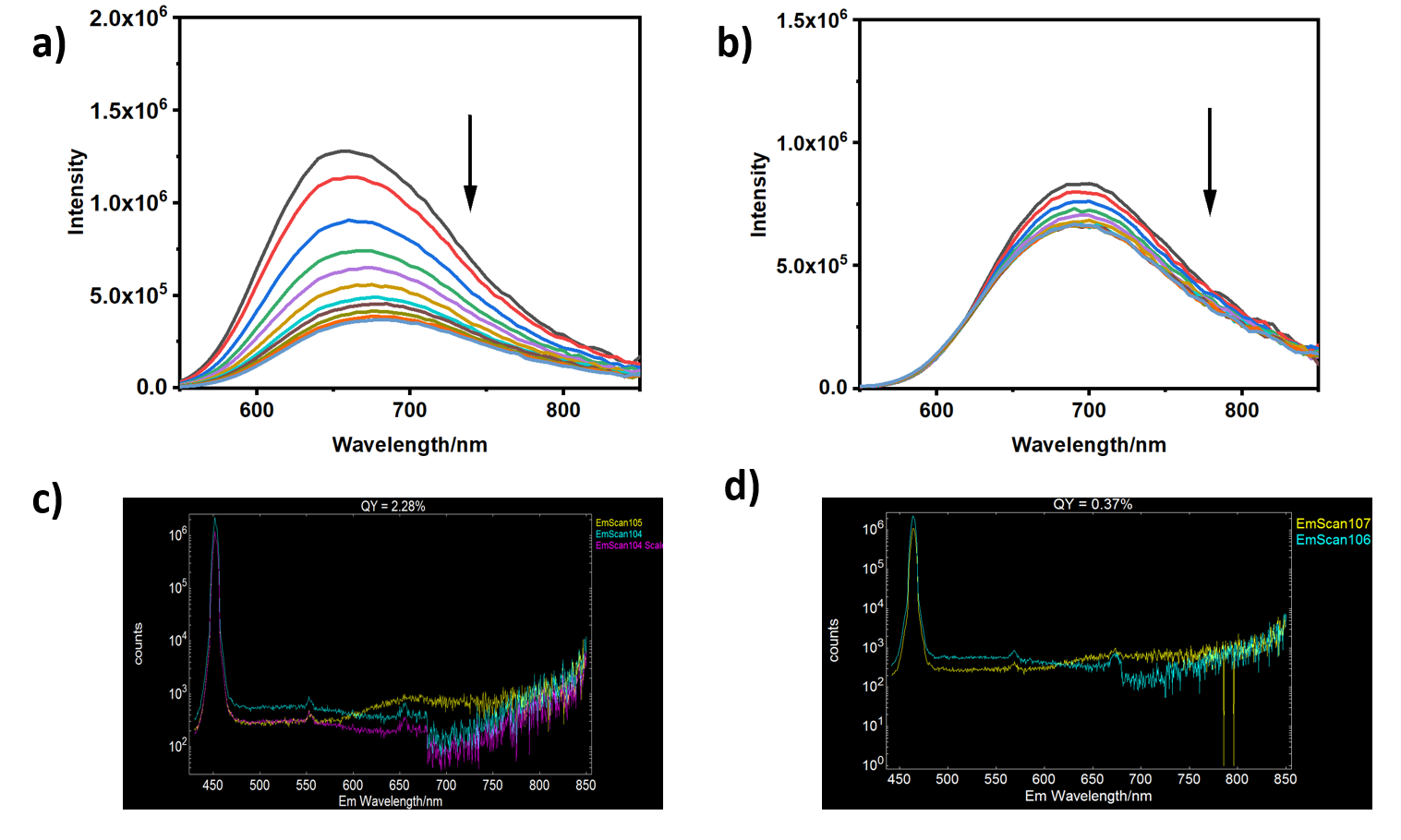


**Figure S41.** a) Fluorescence spectra of G1/CB[7] ([G1] = 0.01 mM, λ_ex_ = 468 nm) and b) G2/CB[7] in the presence of 0-10.0 eq. *L-*FFT ([G2] = 0.01 mM, λ_ex_ = 455 nm). QY of c) G1/CB[7]/*L-*FFT (2.28%) and d) G2/CB[7]/*L-*FFT (0.37%).


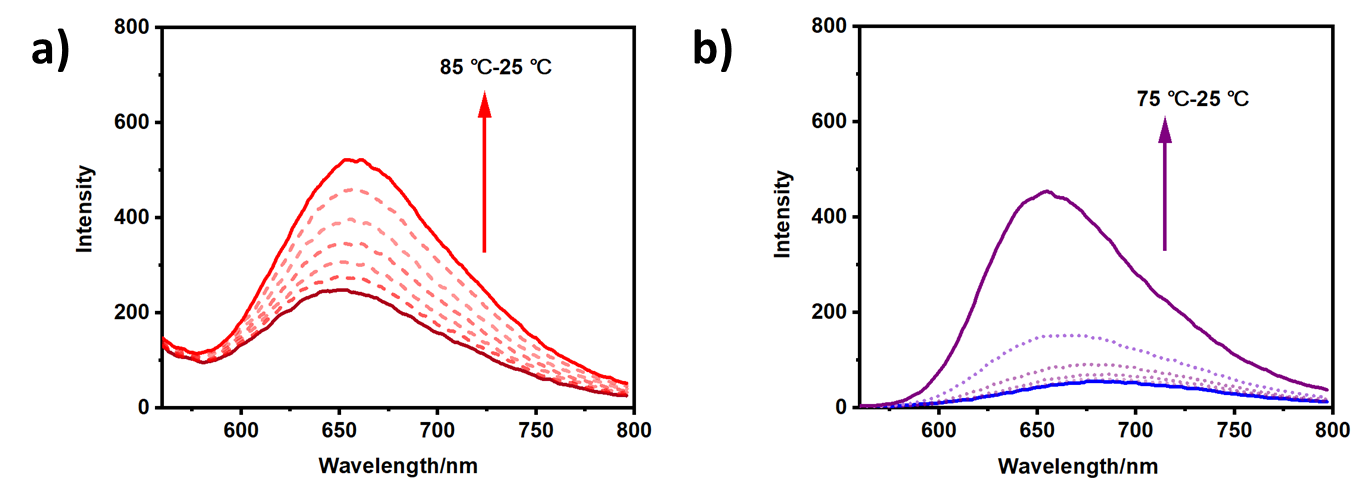


**Figure S42.** Fluorescence spectra of a) G1/CB[8]/*L*-FFT (λ_ex_ = 525 nm, [G1] = 0.01 mM, [CB[8]] = 0.02 mM, [*L*-FFT] = 0.1 mM) and b) G2/CB[8]/*L*-FFT cooled at different temperatures (λ_ex_ = 530 nm, [G2] = 0.01 mM, [CB[8]] = 0.02 mM, [*L*-FFT] = 0.04 mM).


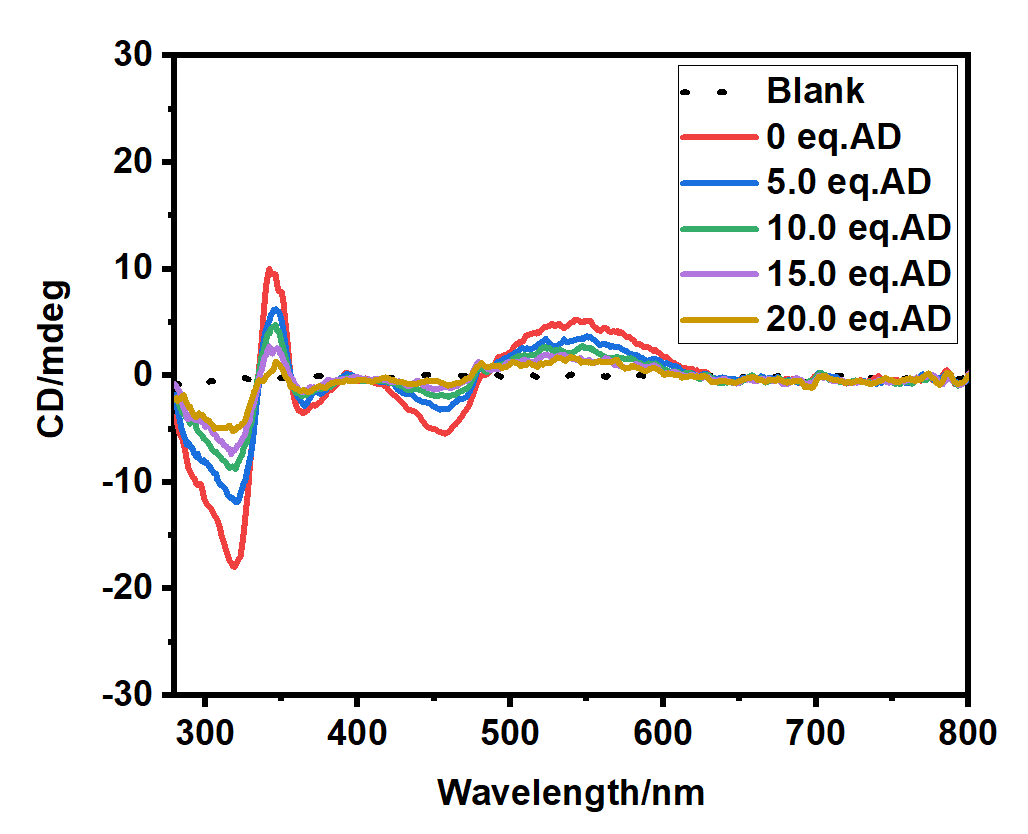


**Figure S43.** The CD spectra of G1/CB[8]/*L*-FFT in the presence of AD ([G1] = 0.05 mM, [CB[8]] = 0.1 mM, [*L*-FFT] = 0.5 mM).

**Cell imaging experiments.**

G2/CB[8] and G2/CB[8]/L-FFT were added to the HeLa cells and further cultured 24 h, after that, the Hochest, or MitoTracker green were added in the above cells, respectively, then washed with PBS for three times, and further observed by confocal laser scanning microscopy.


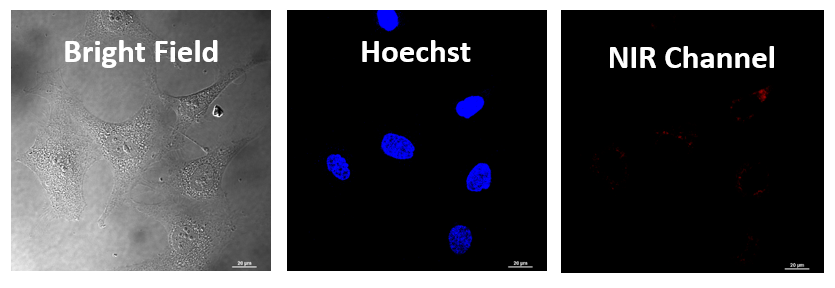


**Figure S44.** Confocal microscopy images of HeLa cells incubated with G2/CB[8] ([G2] = 0.01 mM, [CB[8]] = 0.02 mM).


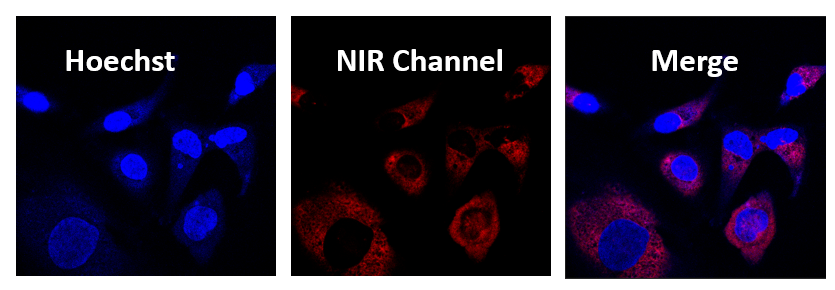


**Figure S45.** Confocal microscopy images of Hela cells incubated with G2/CB[8]/*L*-FFT and Hoechst (a nuclei dye) ([G2] = 0.01 mM, [CB[8]] = 0.02 mM, [*L*-FFT] = 0.04 mM).

Reference:

[1] H. Khelwati, A. W. Franz, Z. Zhou, W. R. Thiel, T. J. J. Müller, *Molecules* **2021**, *26*, 2950.

[2] Frisch, M. J.; Trucks, G. W.; Schlegel, H. B.; Scuseria, G. E.; Robb, M. A.; Cheeseman, J. R.; Scalmani, G.; Barone, V.; Petersson, G. A.; Nakatsuji, H.; Li, X.; Caricato, M.; Marenich, A. V.; Bloino, J.; Janesko, B. G.; Gomperts, R.; Mennucci, B.; Hratchian, H. P.; Ortiz, J. V.; Izmaylov, A. F.; Sonnenberg, J. L.; Williams-Young, D.; Ding, F.; Lipparini, F.; Egidi, F.; Goings, J.; Peng, B.; Petrone, A.; Henderson, T.; Ranasinghe, D.; Zakrzewski, V. G.; Gao, J.; Rega, N.; Zheng, G.; Liang, W.; Hada, M.; Ehara, M.; Toyota, K.; Fukuda, R.; Hasegawa, J.; Ishida, M.; Nakajima, T.; Honda, Y.; Kitao, O.; Nakai, H.; Vreven, T.; Throssell, K.; Montgomery, J. A.; Jr., Peralta, J. E.; Ogliaro, F.; Bearpark, M. J.; Heyd, J. J.; Brothers, E. N.; Kudin, K. N.; Staroverov, V. N.; Keith, T. A.; Kobayashi, R.; Normand, J.; Raghavachari, K.; Rendell, A. P.; Burant, J. C.; Iyengar, S. S.; Tomasi, J.; Cossi, M.; Millam, J. M.; Klene, M.; Adamo, C.; Cammi, R.; Ochterski, J. W.; Martin, R. L.; Morokuma, K.; Farkas, O.; Foresman, J. B.; Fox, D. J. Gaussian 16, Revision A.03; Gaussian, Inc.: Wallingford, CT, 2016.

[3] P. Thordarson, *Chem. Soc. Rev.* **2011**, *40*, 1305-1323.

[4] H. Bakirci, X. Zhang, W. M. Nau, *J. Org. Chem.* **2005**, *70*, 39-46.
